# Dual Sensitization Enables Synergistic Photodynamic Therapy and Radiotherapy for Breast Cancer

**DOI:** 10.34133/research.1114

**Published:** 2026-02-06

**Authors:** Yingying Zhang, Shaoqing Chen, Chun Liu, Pengyin Li, Yutao Zhang, Ziman Li, Xinye Ni

**Affiliations:** ^1^ The Second People’s Hospital of Changzhou, the Third Affiliated Hospital of Nanjing Medical University; Jiangsu Province Engineering Research Center of Medical Physics; Medical Physics Research Center, Nanjing Medical University; Key Laboratory of Medical Physics in Changzhou, Jiangsu, Changzhou 213003, China.; ^2^ School of Pharmacy, Changzhou University, Jiangsu, Changzhou, 213164, China.

## Abstract

Radiotherapy (RT) and photodynamic therapy (PDT) for breast cancer are limited by tumor hypoxia and suboptimal photosensitizer performance. We developed folate-modified copper-doped carbon dots and loaded them with 5-aminolevulinic acid (ALA) to yield FCA, a nanoplatform that executes cascade nanozyme activities to remodel the tumor microenvironment: decomposing H_2_O_2_ to relieve hypoxia, generating hydroxyl radicals and singlet oxygen (^1^O_2_), and depleting glutathione (GSH). This priming enabled efficient ALA-to-protoporphyrin IX conversion, which subsequently amplified reactive oxygen species generation. The elevated oxidative stress then synergized with RT to accumulate DNA double-strand breaks and trigger cell cycle arrest. Consequently, FCA-PDT-RT reduced 4T1 cell viability to 20.09% and induced 83.82% apoptosis outcomes mechanistically linked to nuclear factor erythroid 2-related factor 2 (NRF2)–Kelch-like ECH-associated protein 1 (KEAP1)–heme oxygenase 1 (HMOX1) pathway activation. Despite compensatory upregulation of antioxidant genes (HMOX1 and glutamate–cysteine ligase modifier subunit [GCLM]), intracellular GSH and adenosine triphosphate were severely depleted, establishing a metabolic crisis wherein synthesis could not match consumption. This redox/energy collapse drove the pronounced cytotoxicity observed. In an orthotopic 4T1 model, FCA-PDT-RT achieved superior tumor control at only 12 Gy, which correlated with increased CD3^+^/CD8^+^ T cell infiltration and suppressed angiogenesis, while maintaining favorable safety. FCA thus enables synergistic PDT-RT through sequential microenvironment remodeling, oxidative amplification, and metabolic exhaustion, offering a dose-sparing strategy with translational promise for breast cancer therapy.

## Introduction

Breast cancer remains a leading cause of cancer mortality and is commonly managed with multimodal therapy that includes surgery, systemic agents, and radiotherapy (RT) [1]. As a key component of postoperative adjuvant treatment, RT can eradicate residual local lesions, lower recurrence rates, and prolong survival [2–4]. However, many tumors possess strong DNA repair capacity and antioxidant defenses [5,6], resulting in radioresistance, relapse, and dose-limited toxicities. Strategies that enhance efficacy without dose escalation are therefore an unmet need.

Photodynamic therapy (PDT) generates reactive oxygen species (ROS) upon light activation of a photosensitizer, enabling spatially precise cytotoxicity with minimal systemic exposure and clinical utility in superficial or postsurgical breast lesions [7–9]. However, conventional photosensitizers suffer from limited tissue penetration, modest tumor selectivity, and suboptimal ROS output, while the reductive tumor microenvironment (TME), characterized by elevated glutathione (GSH) and peroxidase (POD) activity, rapidly quenches ROS and blunts efficacy [1]. These barriers have limited broader PDT adoption in breast oncology.

Given their complementary mechanisms, combining PDT with RT has attracted growing interest. Preclinical studies showed that coirradiation can concurrently elevate singlet oxygen (^1^O_2_) and hydroxyl radicals (·OH), depolarize mitochondrial membrane potential (ΔΨm), inactivate respiratory chains, and amplify DNA damage, thereby surpassing additivity and suggesting headroom for dose de-escalation [10–12]. Early translational experiences across several malignancies (e.g., head and neck, lung, prostate, and lymphoma) further indicated feasibility, encouraging responses, and manageable safety when PDT is integrated as salvage, neoadjuvant, or concurrent therapy with RT [13]. Collectively, these data provided a mechanistic and clinical rationale for PDT-RT synergy under clinically relevant, dose-sparing conditions.

Among photosensitizers, 5-aminolevulinic acid (ALA) is a prodrug converted intracellularly to protoporphyrin IX (PpIX), which, upon 660-nm excitation, generates ROS to induce apoptosis and/or necrosis [14–16]. Importantly, PpIX can augment RT-induced oxidative injury and exacerbate DNA and mitochondrial damage, enhancing radiosensitivity [17,18]. These features position ALA as a promising dual-function prodrug for PDT-RT. Nevertheless, ALA’s small size, rapid diffusion, poor tumor selectivity, and swift systemic clearance limit intratumoral PpIX enrichment, motivating dedicated delivery systems that improve pharmacokinetics and on-target conversion in vivo [19,20]. Diverse carriers—including nanoparticles, liposomes, and biomimetic coatings—have increased ALA accumulation and modulated heme biosynthesis to elevate PpIX, illustrating that multidimensional targeting and metabolic pathway control can enhance therapeutic performance [21–23].

Carbon dots (CDs) are attractive delivery scaffolds due to their ultrasmall size, aqueous dispersibility, facile surface functionalization, biocompatibility, and intrinsic fluorescence for imaging [24–26]. Beyond carriage, transition metal doping (e.g., Cu and Mn) can endow CDs with enzyme-mimetic activities—catalase (CAT)-like O_2_ generation via H_2_O_2_ decomposition, oxidase (OXD)-like/POD-like ROS amplification, and glutathione oxidase (GSHox)-like GSH depletion—that remodel the tumor redox milieu. These cascades simultaneously alleviate hypoxia and sustain cytotoxic ROS in antioxidant-rich tumors, thereby potentiating PDT and RT [27–30].

Guided by these considerations, we developed FCA (folate-modified copper-doped CDs [FC] loaded with ALA), a multifunctional nanoplatform that codelivers ALA while executing cascade nanozyme functions within the TME. The copper-doped CDs were synthesized hydrothermally from folic acid and CuCl_2_, followed by electrostatic loading of ALA to obtain FCA. We hypothesized that FCA would (a) enhance tumor accumulation via folate-receptor-mediated targeting, (b) promote intracellular PpIX biosynthesis by improving ALA delivery, and (c) reprogram tumor redox homeostasis through CAT/OXD/POD/GSHox-like activities to concurrently alleviate hypoxia, amplify ROS, and deplete GSH. Collectively, these effects were expected to couple PDT-initiated membrane/mitochondrial injury with RT-induced nuclear DNA damage, thereby yielding true synergistic efficacy under dose-sparing irradiation.

This study therefore integrates codelivery, TME redox modulation, and dual-modality sensitization into a single, targetable nanoplatform for breast cancer. Conceptually, FCA addresses the 2 principal obstacles that limit PDT-RT effectiveness—oxygen shortage and antioxidant quenching—while leveraging the complementary spatial targets of PDT (membrane/mitochondria) and RT (nuclear DNA). Practically, it uses clinically relatable components (ALA, folate receptor targeting, and ultrasmall CDs) and mechanistically coherent redox catalysis to promote sustained oxidative pressure in tumors. We further posited that such redox remodeling would exacerbate replication stress and mitochondrial dysfunction, compromise DNA repair capacity, and ultimately lower the RT dose required for effective tumor control. By establishing a tight mechanistic link between ALA delivery, PpIX enrichment, oxygen/ROS management, and radiosensitization, FCA is designed to advance precise, translational PDT-RT strategies in breast cancer.

## Results and Discussion

### Preparation and characterization of FCA

**Fig. 1.**
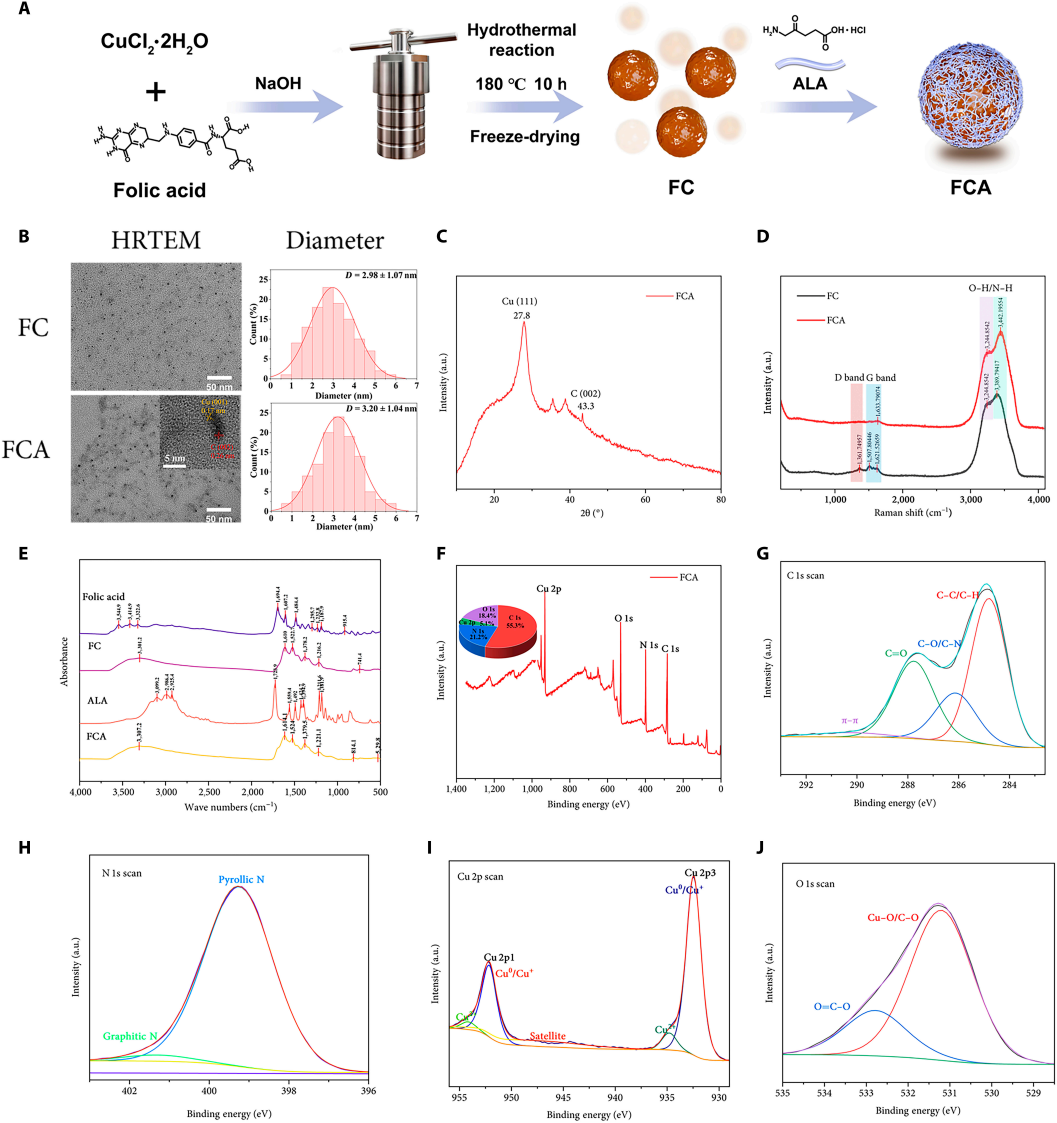
Construction and characterization of the multifunctional FCA nanoplatform. (A) Schematic of FCA synthesis. (B) HRTEM images and size distributions of FC and FCA. Scale bars, 50 nm. (C) XRD pattern of FCA. a.u., arbitrary units. (D) Raman spectra of FC and FCA. (E) FTIR spectra of folic acid, FC, and FCA. (F) XPS survey spectrum of FCA. (G) High-resolution C 1s spectrum of FCA. (H) N 1s spectrum of FCA. (I) Cu 2p spectrum of FCA. (J) O 1s spectrum of FCA.

The preparation process of FCA was illustrated in Fig. 1A. FC were synthesized hydrothermally and subsequently loaded with ALA through electrostatic interactions to generate FCA. High-resolution transmission electron microscopy (HRTEM) revealed monodisperse, quasi-spherical nanoparticles. The mean diameters were 2.98 ± 1.07 nm for FC and 3.20 ± 1.04 nm for FCA, indicating that drug loading did not alter morphology. Distinct lattice fringes with *d* spacings of 0.26 and 0.17 nm can be assigned to the (002) planes of graphitic carbon and the (111) planes of Cu, respectively, suggesting a graphene-like layered framework with embedded Cu nanocrystallites (Fig. 1B). The ALA:FC mass ratio governed payload, which increased from 17.18% to 54.72% across ratios of 0.75 to 2.5 and reached 49.09% at a ratio of 2.0 (Fig. S1); this payload exceeded that of representative lipid nanoparticle and metal–organic framework carriers [31]. On the basis of statistical analysis of the drug loading rates, a mass ratio of 2.0 (ALA-to-FC) was selected for subsequent experiments. Zeta potential decreased from −13.8 ± 1.3 to −23.2 ± 1.1 mV after loading (Fig. S2), consistent with successful assembly and enhanced colloidal stability. X-ray diffraction (XRD) displays a broad graphitic (002) peak and a weak Cu (111) feature, which is further supported by HRTEM lattice fringes of graphitic (002) and Cu (111) (Fig. 1C). Raman spectroscopy exhibited prominent D and G bands, confirming a defect-containing graphitic carbon framework in FCA (Fig. 1D). Fourier transform infrared (FTIR) spectroscopy revealed characteristic surface functional groups and qualitative changes in vibrational features (e.g., aromatic C=C-related bands) compared with folic acid, consistent with carbonization/aromatization (Fig. 1E). X-ray photoelectron spectroscopy (XPS) confirmed a graphitic C 1s envelope, N 1s components consistent with pyridinic and pyrrolic or amine nitrogens, an O 1s distribution indicative of Cu–O and C=O/C–O environments, and a Cu 2p pattern dominated by low-valent copper with a minor Cu^2+^ contribution (Fig. 1F to J) [32]. The ultraviolet-visible (UV-vis) spectrum shows that FCA retains the characteristic absorption features of FC in the UV region (Fig. S3), indicating that ALA loading does not alter the intrinsic optical absorption of the CDs. Consistently, FCA also displayed robust colloidal stability, as its hydrodynamic diameter remained essentially unchanged in saline, 0.5% bovine serum albumin (BSA), and Dulbecco’s modified Eagle’s medium (DMEM) supplemented with 10% fetal bovine serum (FBS) over 1 to 6 d, staying within a narrow range of 10 to 28 nm without a sustained increase (Fig. S4). Notably, the dynamic light scattering (DLS)-derived hydrodynamic size was larger than the TEM size, which can be attributed to the hydrated/solvated shell formed by hydrophilic surface groups on the CDs, further reflecting the favorable aqueous dispersibility and interfacial stability of FCA.

### Multienzyme activities of FCA

**Fig. 2.**
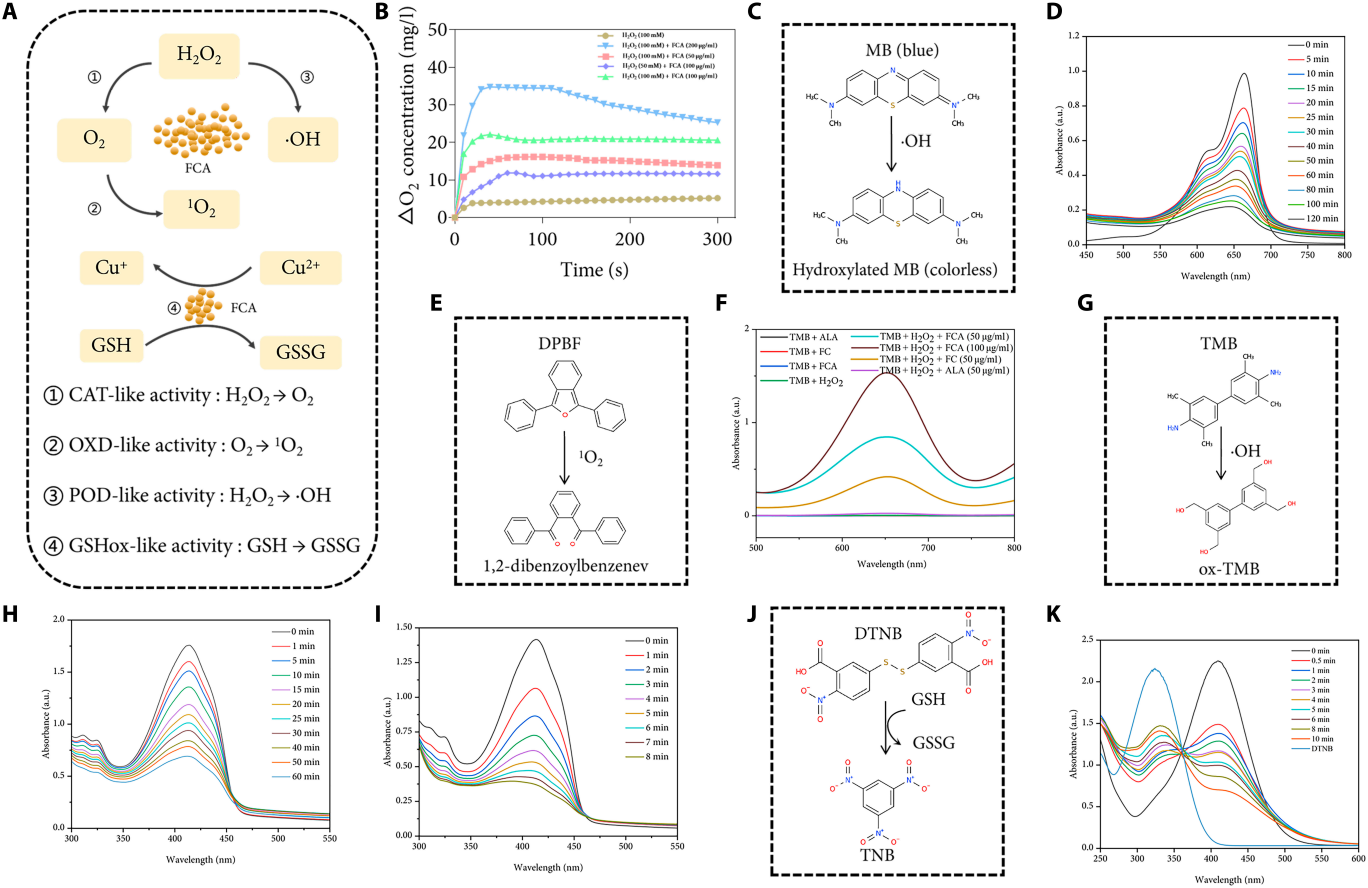
Evaluation of multienzyme mimetic catalytic activities of FCA. (A) Schematic diagram of the multienzyme catalytic mechanism of FCA. GSSG, oxidized GSH. (B) O_2_ generation from H_2_O_2_ catalyzed by FCA. (C) Schematic illustration of ·OH detection using MB. (D) UV-vis absorption spectra showing the degradation of MB by FCA (50 μg/ml) at different time points. (E) Schematic diagram of the oxidation of TMB to oxidized TMB (ox-TMB) by ·OH. (F) Comparison of UV-vis absorption spectra of TMB chromogenic reaction with ALA, FC, and different concentrations of FCA in the presence or absence of H_2_O_2_. (G) Schematic illustration of ^1^O_2_ detection using the DPBF probe. (H) Temporal decay of DPBF absorption monitored by UV-vis spectra during 0 to 60 min of incubation with FCA in the absence of H_2_O_2_. (I) Temporal decay of DPBF absorption monitored by UV-vis spectra during 0 to 8 min of incubation with FCA in the presence of H_2_O_2_. (J) Schematic illustration of GSH depletion detection using the DTNB probe. (K) UV-vis absorption spectra showing the consumption of GSH by FCA monitored by DTNB over 0 to 10 min.

The TME typically contains elevated hydrogen peroxide and GSH, conferring redox advantages to malignant cells [33]. Leveraging copper redox cycling, FCA exhibited a coordinated set of enzyme-mimetic activities that collectively remodel this milieu (Fig. 2A) [34]. First, CAT-like activity decomposed H_2_O_2_ to O_2_ in a substrate- and concentration-dependent manner within FCA (50 to 200 μg/ml) (Fig. 2B), directly addressing oxygen limitation that undermines PDT and contributes to radioresistance. Second, OXD-like and POD-like catalysis produced hydroxyl radicals and singlet oxygen, as validated by methylene blue (MB) decolorization, oxidation of 3,3′,5,5′-tetramethylbenzidine (TMB), and time-resolved quenching of 1,3-diphenylisobenzofuran (DPBF). Across these orthogonal assays, catalytic performance followed the order FCA, FC, and then ALA. The introduction of exogenous H_2_O_2_ accelerated singlet-oxygen generation, indicating a positive feedback between oxygen supply and downstream ROS formation that is conducive to oxidative amplification (Fig. 2C to I) [35]. Third, GSHox-like reactivity rapidly depleted reduced thiols. In a 10 mM GSH system, FCA (10 μg/ml) nearly exhausted GSH within 10 min, demonstrating the capacity to erode the intracellular antioxidant buffer and sustain oxidative pressure (Fig. 2J and K) [36]. These multienzyme-mimetic activities are primarily attributed to the mixed-valence Cu sites (Cu^+^/Cu^2+^) enabling redox cycling and electron transfer, together with the defect-/functional-group-rich CD scaffold that promotes substrate adsorption and interfacial catalysis. The integrated consequence of these activities is conversion of a reductive, therapy-resistant niche into an oxidatively labile state that supported photosensitizer chemistry and radiosensitization.

### Biocompatibility, cellular uptake, and ALA-to-PpIX conversion

**Fig. 3.**
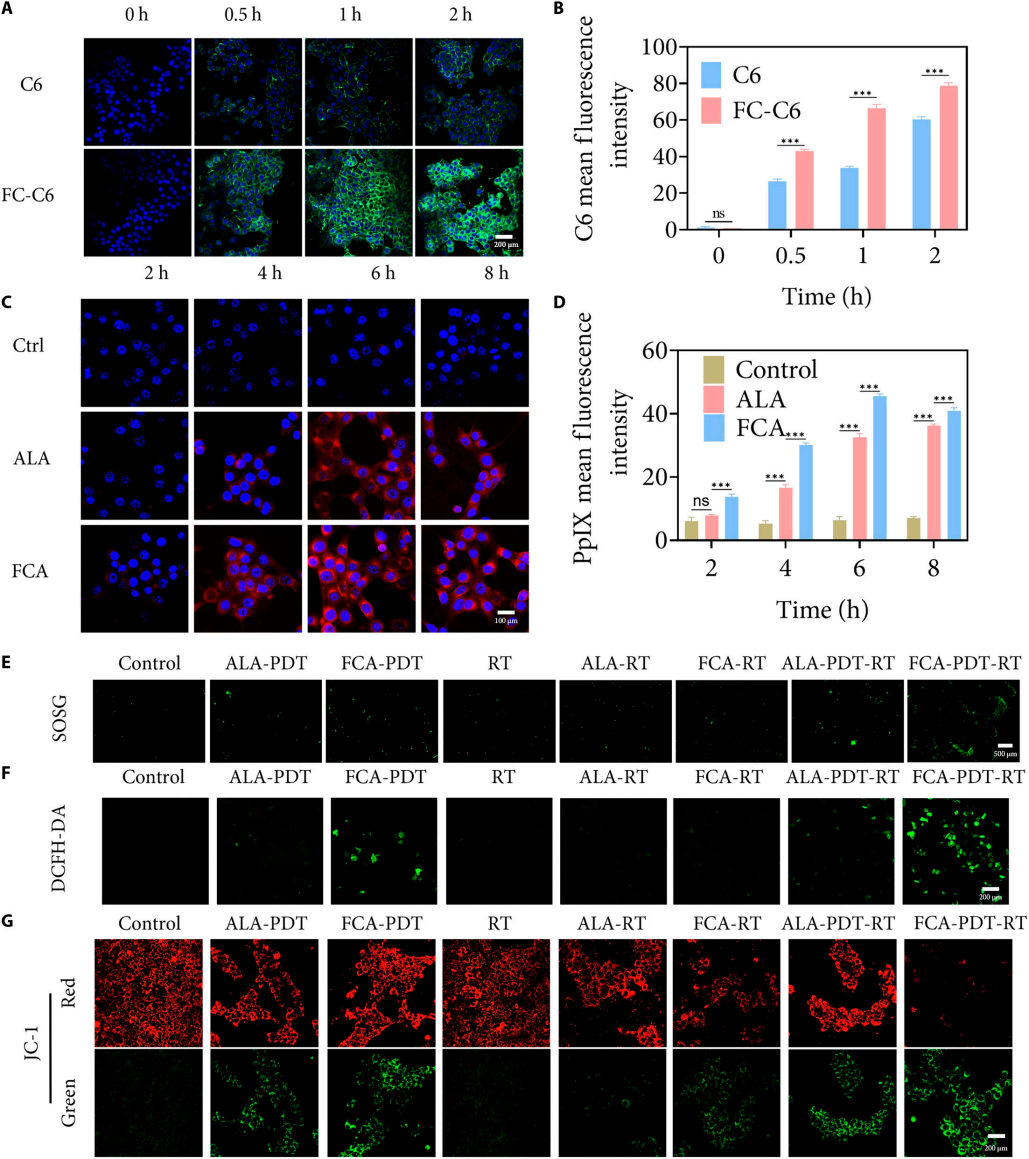
In vitro photodynamic RT synergy and oxidative stress enhancement. (A) Fluorescence images of cellular uptake of free C6 and FC-C6 in 4T1 cells. Green, C6; blue, DAPI nuclear staining. Scale bar, 200 μm. (B) Semiquantitative analysis of cellular uptake kinetics in 4T1 cells. ns, not significant. (C) Fluorescence images of intracellular PpIX in free ALA and FCA groups after different incubation times. Red, PpIX; blue, DAPI nuclear staining. Scale bar, 100 μm. (D) Fluorescence images and semiquantitative analysis of intracellular PpIX in free ALA and FCA groups at different incubation times. (E) SOSG fluorescence images of intracellular ^1^O_2_ in different treatment groups. Green, ^1^O_2_. Scale bar, 500 μm. (F) DCFH-DA fluorescence images of total intracellular ROS in different treatment groups. Green, ROS. Scale bar, 200 μm. (G) JC-1 fluorescence images of ΔΨm in different treatment groups. Red, JC-1 aggregates; green, JC-1 monomers. Scale bar, 200 μm. ns, not significant. ***P < 0.001.

Cytocompatibility screening in MCF-10A, RAW264.7, and 4T1 cells showed viability at or above 80% up to FCA (20 μg/ml) (Fig. S5). Hemolysis remained below 5% across tested concentrations (Fig. S6), supporting acceptable hemocompatibility for systemic use. To assess carrier-mediated internalization, coumarin 6 (C6)-labeled FC (FC-C6) was compared with free C6. Intracellular fluorescence was higher for FC-C6 within 2 h and increased over time, consistent with nanoscale endocytosis and favorable dispersion at the cell interface (Fig. 3A and B) [37]. The enhanced uptake may also reflect increased membrane permeability associated with intracellular hydroxyl radicals produced by copper-mediated Fenton-like reactions under TME-relevant conditions [38]. Because ALA is a prodrug that must be converted to PpIX to act as a photosensitizer, we quantified PpIX fluorescence as a function of incubation time. Free ALA peaked at 6 to 8 h; FCA generated higher PpIX at 6 h and remained elevated thereafter (Fig. 3C and D) [39]. A 6-h interval after administration was therefore selected for all downstream in vitro assays, providing a reproducible window in which the photosensitizer pool and catalytic oxygenation are both favorable.

### Optimizing parameters for FCA-synergized PDT-RT

At a fixed FCA concentration of 20 μg/ml, laser power titration identified 600 mW as the condition that minimized 4T1 viability relative to the no-laser control (Fig. S7A). With laser power set at 600 mW, x-ray dose–response experiments showed a monotonic reduction in viability through 8 Gy (26.6 ± 3.3%), followed by a modest rise at 10 Gy (29.3 ± 1.5%) (Fig. S7B). The latter may reflect shifts in death programs or measurement windows but does not affect the choice of 8 Gy as an effective setting for mechanistic study. The combination of 600 mW and 8 Gy was therefore used in subsequent experiments to evaluate radiosensitization and oxidative coupling.

### ROS and oxidative stress boost in FCA-PDT-RT

Reactive oxygen signaling was quantified with singlet-oxygen sensor green and 2′,7′-dichlorodihydrofluorescein diacetate (DCFH-DA). Under identical irradiation, FCA produced higher singlet oxygen and total ROS than ALA, and coirradiation (PDT together with x-ray) produced the strongest oxidative response (Fig. 3E and F) [40]. These observations accord with a dual-source ROS mechanism in which enriched intracellular PpIX provides a photosensitizer-dependent source upon 660-nm excitation and copper-doped CDs catalytically convert oxygen to singlet oxygen through OXD-like pathways. RT further augmented the FCA-mediated oxidative state during coirradiation, plausibly through mitochondrial stress and facilitated PpIX photochemistry.

### Lipid peroxidation and mitochondrial dysfunction

Malondialdehyde (MDA) is a canonical readout of lipid peroxidation and oxidative membrane damage. As shown in Fig. S8, MDA levels were higher in the FCA-PDT group than in the ALA-PDT group, indicating more extensive peroxidation driven by FCA-induced ROS. The FCA-PDT-RT group showed the greatest increase, supporting an additive/amplifying effect of combined irradiation on membrane injury. ΔΨm was assessed using the JC-1 probe, where a lower red/green fluorescence ratio denotes depolarization and dysfunction [41]. As shown in Fig. 3G and Fig. S9, ΔΨm was lower with FCA-PDT than with ALA-PDT, consistent with stronger ROS-mediated mitochondrial impairment. Under combined treatment, ΔΨm decreased further in the FCA-PDT-RT group and was lower than in FCA-RT and ALA-PDT-RT, indicating that FCA-PDT-RT markedly exacerbates mitochondrial damage and may engage downstream apoptotic signaling.

### Cytotoxicity, cell death, and suppression of proliferation

**Fig. 4.**
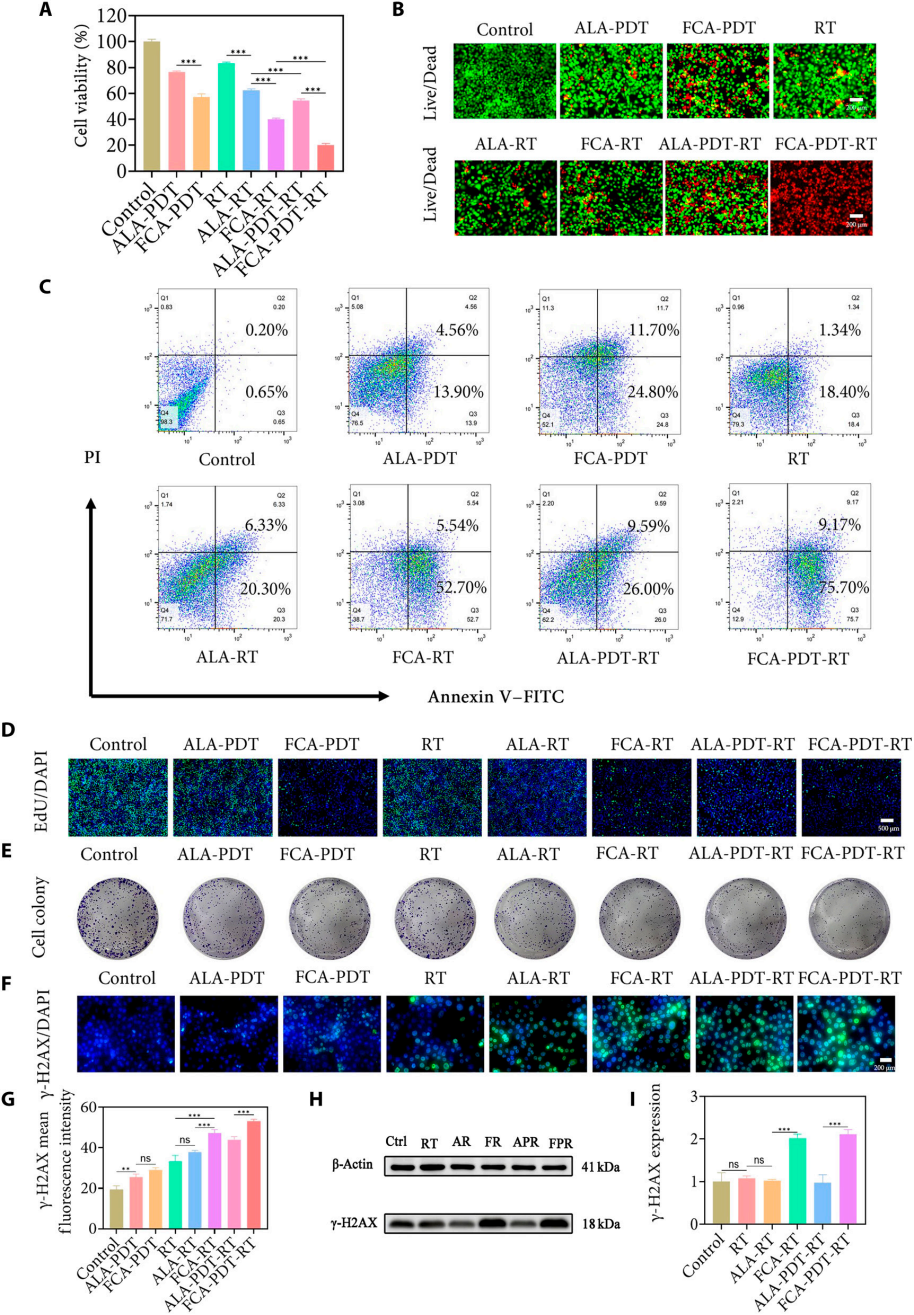
Comprehensive characterization of viability, mode of death, proliferative capacity, and DNA damage in 4T1 cells treated with FCA-synergized PDT-RT. (A) Quantitative CCK-8 results of 4T1 cell viability across treatment groups. (B) Calcein-AM/PI double-stained fluorescence images of 4T1 cells in each treatment group (green, live cells; red, dead cells). Scale bars, 200 μm. (C) Flow cytometric analysis of apoptosis in 4T1 cells stained with annexin V–FITC/PI. (D) EdU incorporation fluorescence images of 4T1 cells (green, EdU-positive proliferating cells; blue, DAPI nuclear staining). Scale bar, 500 μm. (E) Images and statistics of colony formation assay in 4T1 cells across treatment groups. (F) Immunofluorescence images of γ-H2AX staining in 4T1 cells (green, γ-H2AX foci; blue, DAPI). Scale bar, 200 μm. (G) Semiquantitative analysis of γ-H2AX immunofluorescence intensity. (H) Western blot bands of γ-H2AX protein levels in each treatment group. Ctrl, control; AR, ALA-RT; FR, FCA-RT; APR, ALA-PDT-RT; FPR, FCA-PDT-RT. (I) Grayscale value analysis of γ-H2AX protein bands. ns, not significant. **P < 0.01; ***P < 0.001.

A panel of orthogonal assays demonstrated that FCA potentiates both photodynamically induced oxidative injury and radiation-induced genotoxicity. Metabolic viability (Cell Counting Kit-8 [CCK-8]) declined more strongly when ALA was delivered by FCA. Cell death imaging with calcein-acetoxymethyl ester (AM) and propidium iodide (PI) paralleled the metabolic assay, revealing a predominance of nonviable cells under FCA-containing regimens. Annexin V/PI flow cytometry quantified apoptosis and late death phenotypes and showed the strongest effect for the combined FCA-PDT-RT group. Short-term DNA synthesis measured by 5-ethynyl-2′-deoxyuridine (EdU) incorporation and long-term proliferative potential measured by colony formation were both suppressed most severely under the combined regimen (Fig. 4A to E and Figs. S10 to S12) [42]. Quantitatively, combined treatment reduced viability to 20.09% and increased apoptosis to 83.82%, establishing the cooperative benefit of pairing photochemistry with ionizing radiation in the presence of FCA.

### DNA damage and constraint of repair

γ-H2AX immunofluorescence and Western blotting demonstrated accumulation of DNA double-strand breaks. The combined regimen showed the greatest damage magnitude, followed by FCA-RT and then RT alone; FCA-PDT also exceeded ALA-PDT (Fig. 4F to I) [43]. These data aligned with the oxidative measurements and supported an interpretation in which FCA increased both the number and persistence of radiation-induced lesions. Consistently, 8-oxoguanine DNA glycosylase 1 (OGG1) mRNA, a base excision repair factor, was lowest in the combined group (Fig. S13) [44], indicating that DNA damage accrues in a context where a key repair pathway is constrained. The biochemical coupling between oxidative depletion of GSH and repair impairment likely contributed to this phenotype.

### Cell cycle programs and mitochondrial apoptotic signatures

**Fig. 5.**
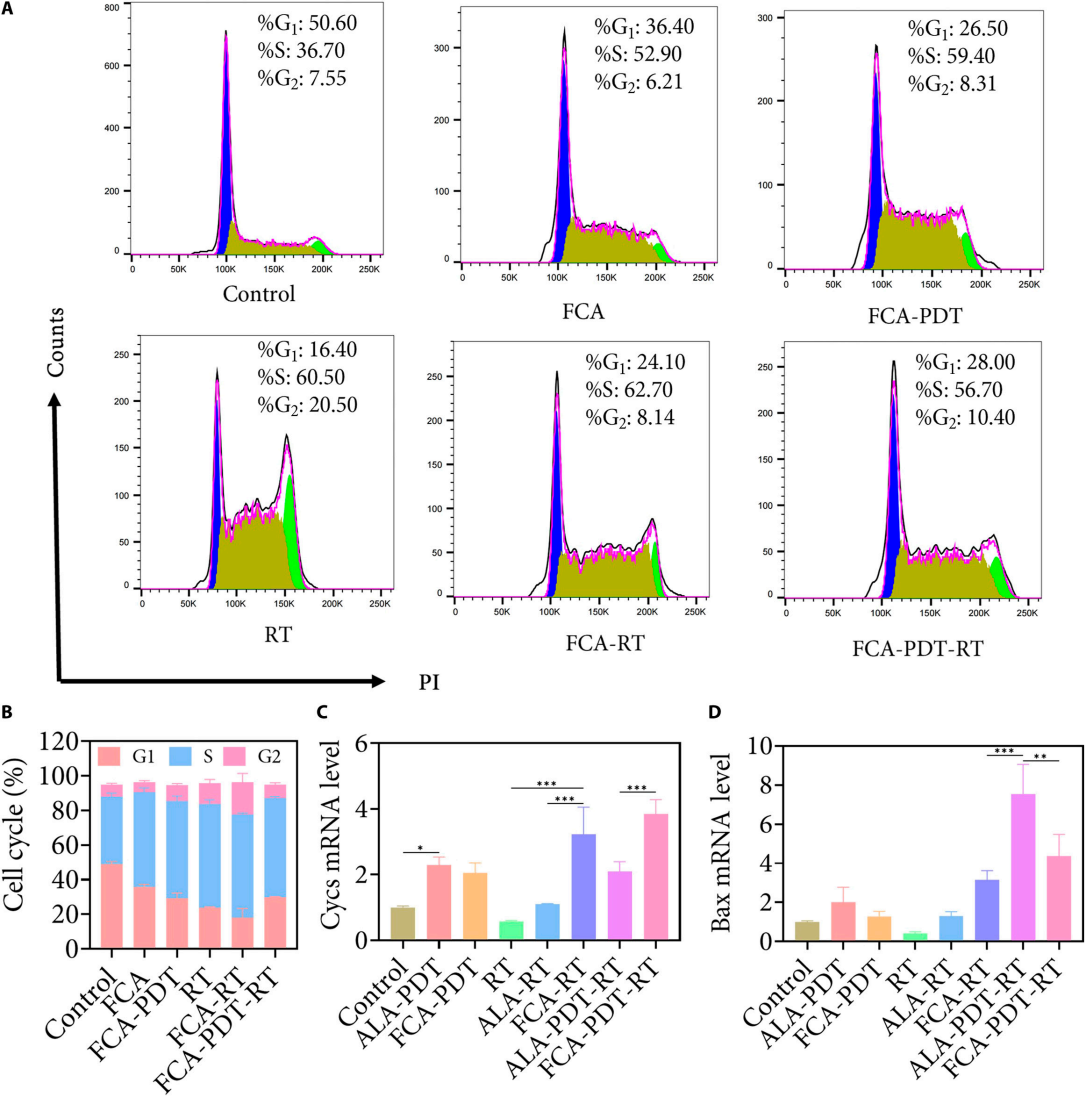
FCA-synergized PDT-RT induces cell cycle arrest and alters expression of apoptosis-related genes. (A) Cell cycle distribution of 4T1 cells in different treatment groups analyzed by flow cytometry. (B) Statistical summary of the proportions of cells in each cycle phase. (C) mRNA expression of cytochrome c (Cycs) in 4T1 cells across treatment groups. (D) mRNA expression of Bax in 4T1 cells across treatment groups. *P < 0.05, **P < 0.01, and ***P < 0.001.

Flow cytometry revealed that FCA alone shifted populations toward S phase accumulation, reflecting replication pressure. RT increased the G_2_-M fraction, consistent with checkpoint activation after DNA damage. The combined FCA-PDT-RT regimen yielded a pattern characterized by a high S phase proportion with a comparatively low G_2_-M proportion, suggesting that replication stress and catastrophic progression contribute more strongly than sustained G_2_-M arrest to cell lethality under combined oxidative and genotoxic insult (Fig. 5A and B). Transcripts associated with mitochondrial apoptosis corroborated this view. Cytochrome c mRNA increased under FCA-PDT and reached its highest level under the combined regimen. Bax mRNA was elevated under both combination regimens, although the absolute transcript level did not linearly predict the magnitude of apoptosis, implying contributions from transcription-independent mechanisms such as mitochondrial permeability transition, posttranscriptional Bax activation and translocation, and attenuation of antiapoptotic proteins (Fig. 5C and D) [45]. Collectively, the cell cycle and apoptosis data indicate that FCA-enabled oxidative stress destabilizes mitochondrial homeostasis and DNA replication, setting the stage for radiosensitized killing.

### Transcriptomics reveal a redox-mitochondrial vulnerability

**Fig. 6.**
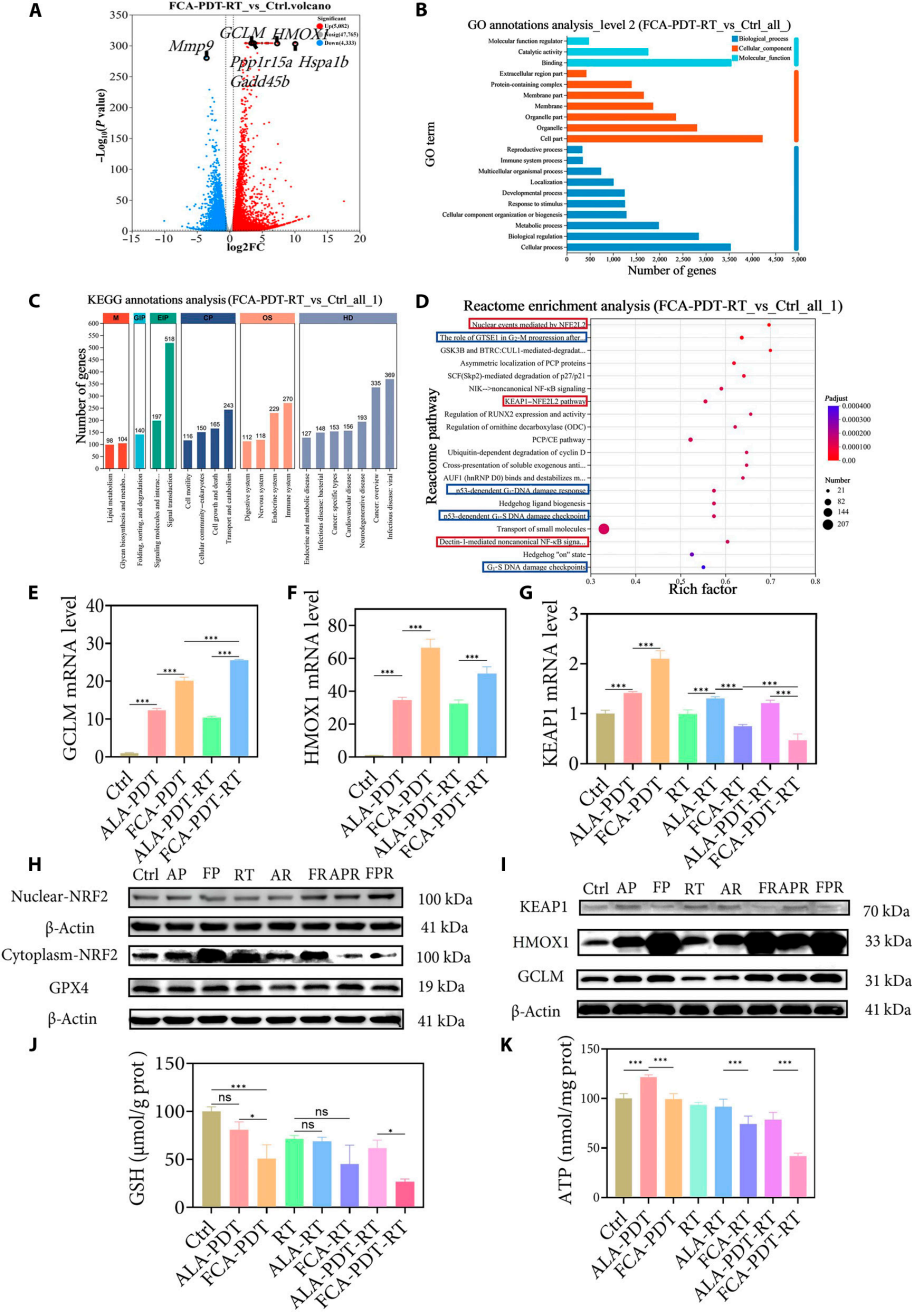
Effects of FCA-mediated combination therapy on the oxidative stress pathway and metabolism in 4T1 cells. (A) Volcano plot of differentially expressed genes between the FCA-PDT-RT group and the control group. Red dots represent up-regulated differentially expressed genes; blue dots represent down-regulated differentially expressed genes. Log_2_FC, log_2_ fold change. (B) Gene Ontology (GO) enrichment analysis of the FCA-PDT-RT group versus the control group. (C) Kyoto Encyclopedia of Genes and Genomes (KEGG) enrichment analysis of the FCA-PDT-RT group versus the control group. (D) Reactome pathway enrichment analysis of the FCA-PDT-RT group versus the control group. The red box highlights the NRF2/HMOX1/KEAP1 pathway; the blue box highlights the cell cycle arrest pathway. (E) mRNA expression levels of the key NRF2 pathway gene GCLM. (F) mRNA expression levels of the key NRF2 pathway gene HMOX1. (G) mRNA expression levels of the key NRF2 pathway gene KEAP1. (H) Western blot analysis of nuclear/cytoplasmic NRF2 and GPX4 protein levels; β-actin was used as the loading control. (I) Western blot analysis of GCLM, HMOX-1, and KEAP1 protein levels in the NRF2 pathway; β-actin was used as the loading control. (J) Intracellular GSH levels in different treatment groups. (K) Intracellular ATP levels in different treatment groups. ns, not significant. *P < 0.05; ***P < 0.001.

To integrate pathway-level responses, we performed RNA sequencing with gene set enrichment. The combined regimen selectively activated the nuclear factor erythroid 2-related factor 2 (NRF2)–Kelch-like ECH-associated protein 1 (KEAP1)–heme oxygenase 1 (HMOX1) axis, indicative of a robust antioxidant response, while broadly down-regulating mitochondrial functional programs, consistent with impaired bioenergetics (Fig. 6A to D and Fig. S14). Quantitative reverse transcription polymerase chain reaction (qRT-PCR) and Western blotting confirmed suppression of KEAP1, nuclear translocation of NRF2, and induction of HMOX1 and glutamate–cysteine ligase modifier subunit (GCLM) (Fig. 6E to I). Despite the transcriptional signature of compensation, intracellular GSH was lowest in the combined group, indicating that consumption exceeded induced synthesis under sustained oxidative pressure (Fig. 6J). In parallel, glutathione peroxidase 4 (GPX4), a regulator of lipid peroxide clearance, was decreased, and adenosine triphosphate (ATP) content declined to approximately 43% of control (Fig. 6K). The conjunction of GSH exhaustion, reduced GPX4, and energy insufficiency established a biochemical context in which antioxidant defenses cannot keep pace with ROS production, thereby explaining the persistence of oxidative damage and the radiosensitized phenotype. The enrichment of NRF2 signaling therefore reflects an attempted but inadequate adaptation to FCA-driven redox stress and mitochondrial dysfunction.

A coherent working model emerged from the multilevel data. FCA concentrated ALA inside tumor cells and supported its conversion to PpIX at a favorable time window. In the TME, FCA supplied oxygen catalytically, amplified ROS generation, and depleted GSH, converting a reductive environment into one that supported oxidative chemistry. The combined result is sustained ROS production under coirradiation, leading to lipid peroxidation, mitochondrial depolarization, and constrained energy metabolism. In this state, regeneration of GSH is limited, and DNA repair is impaired, enabling double-strand breaks to accumulate and replication-associated death to predominate. Mitochondria thus serve as a central damage node that integrates photochemical and ionizing radiation insults, providing a mechanistic basis for the observed synergy.

### In vivo biodistribution

In an orthotopic breast cancer model, free indocyanine green (ICG) or FC-ICG was injected intravenously at 100- to 200-mm^3^ tumor size. In vivo imaging showed minimal tumor signal with free ICG (6 to 48 h), whereas FC-ICG accumulated in tumors in a time-dependent fashion (onset ~ 12 h; pronounced at 24 h; Fig. S15). Ex vivo imaging at 48 h confirmed strong tumor retention only in the FC-ICG group, indicating improved tumor targeting and residence.

### In vivo biocompatibility evaluation

Healthy mice were intravenously injected with FCA or saline via the tail vein and monitored for 12 d. As shown in Fig. S16, body weights in the FCA group remained comparable to controls. Routine blood counts and serum biochemistry (red blood cell [RBC], hemoglobin, platelet, alanine transaminase, and aspartate transaminase) were within reference ranges (Fig. S17). Hematoxylin and eosin (H&E) staining of major organs (heart, liver, spleen, lung, and kidney) revealed no evident histopathological abnormalities or inflammatory lesions (Fig. S18). Collectively, these data indicate good in vivo biocompatibility of FCA over the 12-d observation period.

### Antitumor efficacy with a uniform radiation budget

**Fig. 7.**
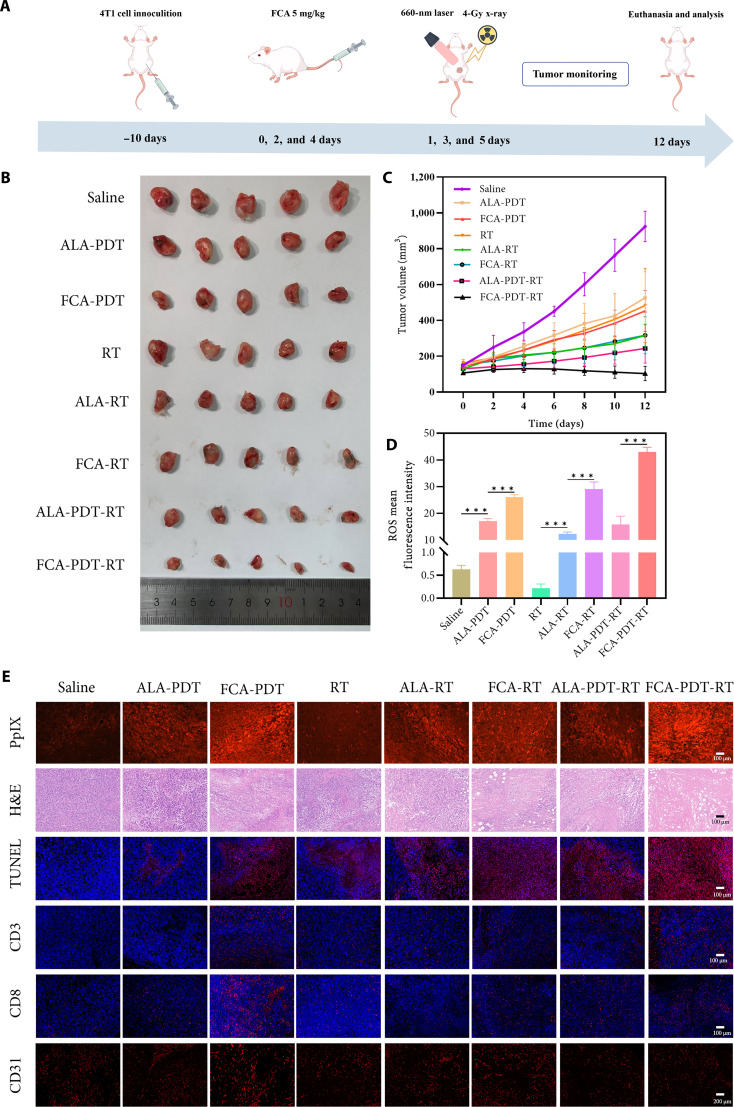
Antitumor efficacy and mechanistic analyses of different treatment strategies in 4T1-tumor-bearing mice. (A) Schematic of 4T1 tumor implantation and treatment schedule. (B) Photographs of excised tumors at the study end point. (C) Tumor growth curves during treatment. (D) Quantification of intratumoral ROS levels (bar graph). (E) Multiplex immunofluorescence and H&E staining of tumor sections. PpIX staining, red fluorescence that indicates PpIX signal (scale bar, 100 μm); H&E, representative histomorphology (scale bar, 100 μm); TUNEL, TUNEL-positive (apoptotic) cells (red) and DAPI-stained nuclei (blue) (scale bar, 100 μm); CD3 immunofluorescence, CD3^+^ T cells (red) and DAPI-stained nuclei (blue) (scale bar, 100 μm); CD8 immunofluorescence, CD8^+^ cytotoxic T cells (red) and DAPI-stained nuclei (blue) (scale bar, 100 μm); CD31 immunofluorescence, CD31^+^ vascular endothelial cells (red) (scale bar, 100 μm). ***P < 0.001.

Mice were randomized to 8 groups (*n* = 5 per group): saline, ALA-PDT, FCA-PDT, RT, ALA-RT, FCA-RT, ALA-PDT-RT, and FCA-PDT-RT. The cumulative x-ray dose was held constant at 12 Gy across all in vivo regimens. Over a 12-d observation period, saline-treated tumors expanded by 6.8-fold, whereas the combined FCA-PDT-RT group limited growth to 1.5-fold (Fig. 7A to C). The end-point tumor weight was lowest in the combined group, consistent with volumetric trends (Fig. S19). FCA-RT inhibited growth more effectively than RT alone, and FCA-PDT surpassed ALA-PDT, supporting the conclusion that FCA confers dual sensitization. Body weight remained stable during treatment, and H&E staining of the heart, liver, spleen, lung, and kidney revealed no overt lesions under FCA-containing regimens, comparable to saline (Figs. S20 and S21). These findings substantiate therapeutic benefit at a radiation dose consistent with dose-sparing objectives.

### In-vivo correlates of synergy

Mechanistic measurements in vivo mirrored the in vitro observations. Flow cytometry showed stepwise increases in intratumoral ROS from ALA-PDT to FCA-PDT and to the combined regimen; the combined group exhibited a 2.7-fold increase relative to the ALA-based combination (Fig. 7D and Fig. S22). PpIX imaging was weak in ALA-based regimens, increased with FCA delivery, and was maximal under the combined regimen (Fig. 7E). Histology revealed extensive coagulative necrosis with narrow rims of viable tissue in the combined group at a total x-ray dose of 12 Gy; FCA-PDT and FCA-RT produced larger necrotic areas than their ALA counterparts. Terminal deoxynucleotidyl transferase–mediated deoxyuridine triphosphate nick end labeling (TUNEL) staining showed the most abundant apoptosis in the combined group. Immune profiling indicated increased CD3^+^ T cell infiltration with FCA-PDT and with FCA-RT and the highest infiltration with the combined regimen; CD8^+^ cytotoxic T cells were more abundant than in ALA-based combinations; and CD31 staining was lowest in the combined group, indicating attenuated angiogenesis. These data suggest that oxidative priming and mitochondrial injury cooperate with radiogenic DNA damage to remodel both tumor and stromal compartments.

**Fig. 8.**
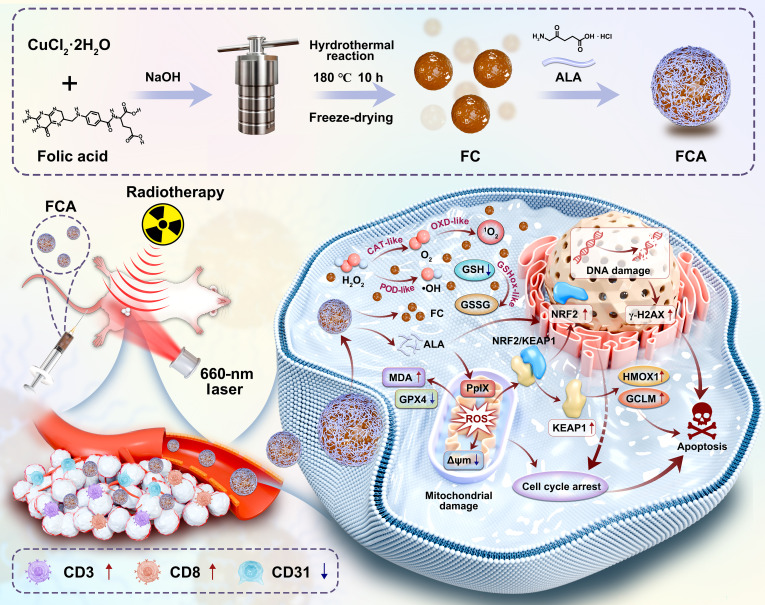
Schematic illustration of the multifunctional nanoplatform FCA for synergistic sensitization in combined therapy.

Overall, by enhancing tumor-targeted accumulation, amplifying oxidative stress, promoting immune infiltration, and suppressing angiogenesis, FCA achieves synergistic potentiation of PDT and RT in vivo, demonstrating robust multimodal therapeutic potential with favorable biosafety (Fig. 8).

## Conclusion

In this study, we engineered FCA, TME catalysis, and dual-modality sensitization for PDT and RT. FCA is small (~3 nm), biocompatible, and supported high ALA payloads (49.09% at ALA:FC = 2.0), facilitated efficient intratumoral delivery. In the TME, FCA showed CAT/POD/OXD/GSHox-like activities, which supply O_2_, amplify ROS, and deplete GSH; these effects enriched PpIX and increased ^1^O_2_ for PDT while cooperated with RT-induced double-strand breaks. This yielded lipid peroxidation, ΔΨm depolarization, cytochrome-c-mediated apoptosis, and S phase accumulation with disturbed G_2_-M features of replication-associated lethality. Transcriptomics and validation indicated NRF2–KEAP1–HMOX1 activation with redox decompensation (GSH exhaustion, ATP decline, and mitochondrial program down-regulation), further sensitizing tumors to PDT and RT. In vivo, FCA enhanced tumor accumulation, increased intratumoral PpIX/ROS, augmented CD3^+^/CD8^+^ infiltration, suppressed CD31^+^ angiogenesis, and maintained systemic safety; notably, near-ablative control was achieved with only 12-Gy total irradiation, underscoring dose-sparing PDT-RT synergy. Notably, the 12-Gy (4 Gy × 3) regimen in mice is used as a reduced RT input to enable quantification of radiosensitization rather than to directly match clinical physical doses; the observed efficacy gain supports the feasibility of RT de-escalation in future translational settings. FCA thus offers a mechanistically coherent and translational route to precise PDT-RT combination therapy, with rationale for integration with immune checkpoint blockade.

## Materials and Methods

### Materials

Folic acid (97%; high-performance liquid chromatography grade), copper(II) chloride dihydrate (analytical reagent [AR] grade), ALA hydrochloride (99%), ethyl acetoacetate (99%; gas chromatography grade), iron(III) chloride hexahydrate (99%; AR grade), MB (70%), DPBF (97%), GSH (98%), 5,5′-dithiobis(2-nitrobenzoic acid) (DTNB; 98%), C6 (98%), and the singlet-oxygen probe SOSG (singlet-oxygen sensor green) were purchased from Aladdin Reagent Co. Ltd. (Shanghai, China).

TMB (99%) was obtained from Macklin Reagent Co. Ltd. (Shanghai, China). Sodium hydroxide was supplied by Sinopharm Chemical Reagent Co. Ltd. (Shanghai, China). Dialysis bags with a molecular weight cutoff of 3.5 kDa were obtained from Yuanye Bio-Technology Co. Ltd. (Shanghai, China).

FBS was obtained from Thermo Fisher Scientific Gibco (Waltham, MA, USA). Penicillin–streptomycin solution (100×), 0.25% Trypsin-EDTA, phosphatase inhibitor cocktail I (100×), 5× SDS-polyacrylamide gel electrophoresis (PAGE) sample loading buffer, bicinchoninic acid protein assay kit, DNA damage assay kit, EdU-488 cell proliferation assay kit, crystal violet staining solution, cell cycle and apoptosis analysis kit, enhanced ATP assay kit, and BeyoECL Plus chemiluminescent substrate were all purchased from Beyotime Biotechnology (Beijing, China). DMEM and the CCK-8 enhanced solution were supplied by Meilun Biotechnology Co. Ltd. (Dalian, China). Phosphate-buffered saline (PBS) (1×, pH 7.4) was obtained from Servicebio (Wuhan, China). Deoxyribonuclease I and Liberase TL were purchased from Roche (Basel, Switzerland). The ΔΨm probe JC-1 was purchased from Thermo Fisher Scientific (Waltham, MA, USA). The MDA Assay Kit was obtained from Solarbio Science & Technology Co. Ltd. (Beijing, China). The Live & Dead Viability/Cytotoxicity Assay Kit for animal cells was purchased from UElandy (Suzhou, China). The GSH Assay Kit was supplied by KeyGEN BioTECH (Nanjing, China). The RNApure Fast Tissue & Cell Kit (catalog no. CW0599S) was obtained from ComWin Biotech Co. Ltd. (Beijing, China). HiScript II Q RT SuperMix for qPCR was purchased from Tsingke Biotechnology Co. Ltd. (Shanghai, China). The 2× Realab Green PCR Fast Mixture was obtained from LABLEAD (Beijing, China). The One-Step PAGE Gel Fast Preparation Kit (12%) was purchased from Vazyme Biotech Co. Ltd. (Nanjing, China). The Prestained Protein Marker (Dual Color) was sourced from Epizyme Biomedical Technology Co. Ltd. (Shanghai, China). The QuickBlock Rapid Blocking Buffer (instant granules) was purchased from ShareBio (Hangzhou, China). The anti-γ-H2AX antibody was obtained from Abcam (Cambridge, UK), and antibodies against β-actin, NRF2, GPX4, KEAP1, HMOX1, and GCLM were purchased from Wuhan San Ying Biotechnology Co. Ltd. (Wuhan, China).

Unless otherwise specified, all other chemical reagents were of chromatographic or analytical grade and were used without further purification.

### Synthesis of FC

Folic acid (120 mg) was dispersed in deionized water (60 ml), and 1 M NaOH was added dropwise until complete dissolution. CuCl_2_·2H_2_O (40 mg) was added, the pH was adjusted to ~7, and the mixture was transferred into a Teflon-lined autoclave and heated at 180 °C for 10 h. After cooling, suspensions were centrifuged at 5,000 rpm for 10 min to remove aggregates, followed by 12,000 rpm for 30 min. The precipitate was washed with water and lyophilized to obtain FC.

### Preparation of FCA, FC-C6, and FC-ICG

To prepare the ALA-loaded nanocomplex (FCA), ALA was mixed with FC at various mass ratios (ALA:FC = 0.75:1, 1:1, 1.25:1, 1.5:1, 1.75:1, 2:1, and 2.25:1), respectively. The mixtures were stirred (200 rpm) in the dark at 37 °C overnight. The mixtures were dialyzed (molecular weight cutoff, 3.5 kDa) against deionized water for 24 h to remove free ALA and then freeze-dried to yield FCA.

FC-C6 and FC-ICG were prepared similarly. Briefly, C6 or ICG was mixed with FC at a mass ratio of 2:1 (C6/ICG:FC). The mixtures were stirred, dialyzed, and lyophilized using the same conditions described above for FCA.

### Determination of ALA loading efficiency

Free ALA in dialysate was determined by a modified Ehrlich colorimetric assay. Briefly, dialysate was reacted with ethyl acetoacetate and sodium acetate–acetic acid buffer at 100 °C, followed by HCl and FeCl_3_ treatment and reheating. After cooling, absorption at 470 nm was recorded and ALA content was calculated from a calibration curve. Loading efficiency was calculated as follows:

$$\begin{array}{c} \mathrm{ALA}\;\text{Loading Efficiency}\;\left(\%\right)=\\ \left[\left(\;{\mathrm{ALA}}_{\text{total}}-{\mathrm{ALA}}_{\text{free}}\right)/{\mathrm{ALA}}_{\text{total}}\right]\times 100\% \end{array}$$ (1)

### Characterization of FCA

Morphology and lattice fringes were observed by HRTEM (Tecnai G20, FEI, USA). The hydrodynamic diameter and zeta potential were measured by DLS using a Zetasizer Nano instrument (Malvern Panalytical, UK). The crystal structure was characterized by Raman spectroscopy (DXR, Thermo Fisher Scientific, USA) and XRD (X’Pert PRO MPD, Malvern Panalytical, The Netherlands). Elemental composition and chemical states were analyzed by XPS (Thermo K-Alpha, Thermo Fisher Scientific, USA). Functional groups were identified by FTIR spectroscopy (Nicolet iS 10, Thermo Fisher Scientific, USA).

### UV-vis absorption spectroscopy of FCA

ALA, FC, and FCA were each dispersed in deionized water. UV-vis absorption spectra were recorded using a UV-vis spectrophotometer over the wavelength range of 200 to 800 nm. The spectra were collected and compared to evaluate the characteristic UV region absorption features of ALA, FC, and FCA.

### Hydrodynamic size stability of FCA

Three media were prepared: physiological saline (0.9% NaCl), 0.5% BSA solution, and complete DMEM supplemented with 10% FBS. FCA was dispersed in each medium. The samples were incubated at 37 °C under static conditions, and aliquots were collected on days 0, 1, 2, 4, and 6 for measurement of the hydrodynamic diameter using DLS.

### Validation of CAT-like activity

FCA (50 to 200 μg/ml) was added to PBS (pH 7.4) containing 50 or 100 mM H_2_O_2_. Dissolved oxygen was monitored every 10 s for 5 min with a portable oxygen meter.

### Validation of POD-like activity

For MB bleaching, FCA (50 μg/ml) was mixed with MB (0.015%) and H_2_O_2_ (10 mM), and absorbance at 650 nm was followed up to 120 min. For TMB oxidation, FC or FCA (50 or 100 μg/ml, FC-equivalent) was incubated with TMB (10 mM) and H_2_O_2_ (10 mM) in PBS for 30 min, and oxidized TMB was measured at 652 nm.

### Validation of OXD-like activity

FCA (20 μg/ml) was incubated with DPBF (0.35 mM), and the decrease in absorbance at 415 nm was recorded. For H_2_O_2_-assisted reactions, FCA and H_2_O_2_ (10 mM) were premixed before DPBF addition, and absorbance at 415 nm was monitored for 0 to 8 min.

### Validation of GSHox-like activity

FCA (20 μg/ml) and GSH (200 μM) were incubated in PBS (pH 7.4). At defined times, DTNB was added, and absorbance at 412 nm was measured to evaluate GSH consumption.

### Hemolysis assay

RBCs from Balb/c mice were washed and diluted in saline to 2% (v/v). FCA (12.5 to 100 μg/ml) was incubated with the RBC suspension at 37 °C for 2 h. PBS and 1% Triton X-100 were used as negative and positive controls. After centrifugation, supernatants were measured at 540 nm, and hemolysis percentage was calculated relative to the controls. The hemolysis ratio (HR) was calculated as follows:

$$\mathrm{HR}\;\left(\%\right)=\left[\left(A_{s}-A_{n}\right)/\left(A_{p}-A_{n}\right)\right]\times 100\%$$ (2)

where *A*_s_, *A*_n_, and *A*_p_ are the absorbances of the sample, negative control, and positive control, respectively.

### Cell culture

The human mammary epithelial cell line MCF-10A, the murine monocyte/macrophage cell line RAW264.7, and the murine breast cancer cell line 4T1 were purchased from Zhong Qiao Xin Zhou Biotechnology (Shanghai, China). All cells were cultured in DMEM supplemented with 10% FBS and 1% penicillin–streptomycin solution at 37 °C in a humidified atmosphere containing 5% CO_2_ (Thermo Fisher Scientific, USA).

### Biocompatibility of FCA

For cytocompatibility evaluation, cells were seeded in 96-well plates (1 × 10^4^ cells per well), cultured for 12 h, and then incubated with FCA (0 to 60 μg/ml) for 24 h. CCK-8 solution was added, and absorbance at 450 nm was used to calculate relative viability. Cell viability was calculated as follows:

$$\text{Cell Viability}\;\left(\%\right)=\left[\left(A_{s}-A_{b}\right)/\left(A_{c}-A_{b}\right)\right]\times 100\%$$ (3)

where *A*_s_, *A*_c_, and *A*_b_ represent the absorbance of the sample, control (untreated cells), and blank (medium only) wells, respectively.

### Cellular uptake and PpIX generation

For uptake, 4T1 cells were grown on glass-bottom dishes and incubated with free C6 or FC-C6 (C6, 19.6 μg/ml) for up to 2 h. After fixation with 4% paraformaldehyde and 4′,6-diamidino-2-phenylindole (DAPI) staining, and confocal laser scanning microscopy (ZEISS LSM 900, Germany), images were acquired and analyzed by ImageJ.

For PpIX bioconversion, 4T1 cells were treated with ALA or FCA (ALA-equivalent 19.6 μg/ml) for 2 to 8 h, fixed, and stained with DAPI, and PpIX fluorescence was quantified from confocal laser scanning microscopy (CLSM) images.

### Phototoxicity under different irradiation conditions

To assess laser-power-dependent toxicity, 4T1 cells were pretreated with FCA (20 μg/ml) for 6 h, irradiated with a 660-nm laser (0 to 800 mW for 10 min), and analyzed by CCK-8 after 24 h.

For radiation dose dependence, FCA-pretreated cells received 660-nm laser irradiation (600 mW for 10 min; laser-to-sample distance, 10 cm), followed by 6-MV x-ray exposure (0 to 10 Gy; source-to-sample distance, 100 cm) using a clinical linear accelerator, and cell viability was measured 24 h later.

### Intracellular ^1^O_2_ and ROS

Singlet oxygen was detected using SOSG. 4T1 cells were treated with ALA or FCA (ALA-equivalent, 19.6 μg/ml) for 6 h, incubated with SOSG, and subjected to 8-Gy x-rays or 660-nm laser irradiation (600 mW for 10 min). After 30 min, cells were fixed and counterstained with DAPI for fluorescence imaging.

Total ROS was quantified with DCFH-DA. 4T1 cells were treated as above, incubated with DCFH-DA (40 μM) for 20 min, irradiated (8-Gy x-rays or 660-nm laser), and incubated for 1 h. Fluorescence images were collected, and mean intensity was calculated.

### Lipid peroxidation and ΔΨm

For MDA, 4T1 cells (1 × 10^5^ cells per well) were treated with ALA or FCA (ALA-equivalent, 19.6 μg/ml), irradiated (8-Gy x-rays or 660-nm laser), and incubated for 24 h. Cell lysates were analyzed with an MDA kit and normalized to protein content.

ΔΨm was measured with JC-1. After treatment and irradiation, 4T1 cells were stained with JC-1 at 37 °C for 30 min and observed by CLSM. The red/green fluorescence ratio was used to evaluate ΔΨm disruption.

### Combination therapy in vitro

For PDT-RT combination, 4T1 cells in 96-well plates were incubated with ALA or FCA (ALA-equivalent, 19.6 μg/ml) for 6 h, irradiated with 660-nm laser (600 mW for 10 min) and/or 8-Gy x-rays, and cultured for 24 h. CCK-8 assays quantified cell viability.

Live/Dead staining was performed on similarly treated cells in 6-well plates using calcein-AM/PI.

Apoptosis was analyzed by annexin V–fluorescein isothiocyanate (FITC)/PI staining and flow cytometry.

### Proliferation, clonogenic survival, and DNA damage

EdU incorporation was used to assess proliferation. After treatments, 4T1 cells were incubated with EdU (20 μM) for 2 h, fixed, and processed according to the kit protocol, followed by DAPI staining. EdU-positive cells were counted from fluorescence images.

For colony formation, 1 × 10^3^ 4T1 cells per well were seeded in 6-well plates, treated with ALA or FCA-based PDT and/or RT, and then cultured in fresh medium for ~7 d. Colonies were fixed, stained with crystal violet, and counted.

γ-H2AX immunofluorescence was used to evaluate DNA double-strand breaks. Two hours after irradiation, treated cells were fixed, incubated with γ-H2AX antibody and fluorescent secondary antibody, counterstained with DAPI, and imaged. Foci per nucleus were quantified.

### Cell cycle, GSH, and ATP

For cell cycle analysis, treated 4T1 cells were fixed in 75% ethanol at 4 °C overnight, stained with PI, and analyzed by flow cytometry.

Intracellular GSH and ATP were measured from cell lysates obtained 24 h after treatment, using commercial kits and normalizing to total protein.

### RNA sequencing and qRT-PCR

For RNA sequencing, 4T1 cells in 10-cm dishes were treated with FCA (20 μg/ml) plus PDT and/or RT. After 24 h, total RNA was extracted, polyadenylate-enriched, used for library preparation, and sequenced on an Illumina platform. Clean reads were aligned to the reference genome, and differential expression was analyzed with DESeq2. For qRT-PCR, total RNA from treated 4T1 cells was reverse-transcribed to cDNA. SYBR green-based qPCR was performed with glyceraldehyde-3-phosphate dehydrogenase as the reference gene. Relative expression was calculated by the 2^−∆∆Ct^ method. The primers used in the experiment are listed in Table 1.

**Table 1.** Primer sequences used for qRT-PCR in 4T1 cells

Gene	5′–3′	3′–5′
HMOX1	CACTCTGGAGATGACACCTGAG	GTGTTCCTCTGTCAGCATCACC
GCLM	AGGAGCTTCGGGACTGTATCC	GGGACATGGTGCATTCCAAAA
KEAP1	ATCCAGAGAGGAATGAGTGGCG	TCAACTGGTCCTGCCCATCGTA
Cyclin B	AAGGTGCCTGTGTGTGAACC	GTCAGCCCCATCATCTGCG
Cycs	CCAAATCTCCACGGTCTGTTC	ATCAGGGTATCCTCTCCCCAG
OGG1	CTGCCTAGCAGCATGAGACAT	CAGTGTCCATACTTGATCTGCC

### Western blot

Treated 4T1 cells were lysed in radioimmunoprecipitation assay buffer with protease/phosphatase inhibitors. Equal amounts of protein were separated by SDS-PAGE and transferred to polyvinylidene difluoride membranes. After blocking, membranes were incubated with primary antibodies (γ-H2AX, NRF2, GPX4, KEAP1, HMOX1, GCLM, and β-actin) and secondary antibodies. Bands were visualized by enhanced chemiluminescence.

### Animal studies, procurement, and housing

Female Balb/c mice (6 to 8 weeks old) were supplied by Jiangsu Changzhou Cavens Experimental Animal Co. Ltd. All animal experiments were approved by the Institutional Animal Care and Use Committee (IACUC) of Jiangsu Kebiao Medical Testing Co. Ltd., with the IACUC review number: IACUC25-0227. Mice were housed under specific-pathogen-free conditions (temperature, 25 ± 2 °C; humidity, 50 ± 5%) with free access to autoclaved food and water. All procedures were conducted in accordance with relevant national and international guidelines.

### In vivo biodistribution

Orthotopic 4T1 tumors were generated by injecting 1 × 10^7^ cells (100 μl) into the mammary fat pad. When tumors reached ~100 mm^3^, mice received intravenous injection of free ICG or FC-ICG (50 μl, 1 mg/ml, ICG-equivalent). Whole-body near-infrared fluorescence imaging was conducted at 6, 12, 24, and 48 h. At 48 h, mice were euthanized, and major organs and tumors were collected for ex vivo imaging.

### In vivo biocompatibility evaluation of FCA

Healthy mice were randomly assigned to a saline group or an FCA group (*n* = 3). Mice were intravenously injected via the tail vein with 50 μl of saline or FCA (1 mg/ml). The injections were administered 3 times, and body weight was recorded every other day. On day 12, mice were euthanized and blood samples were collected for serum biochemistry and routine hematology analyses. Meanwhile, major organs (heart, liver, spleen, lung, and kidney) were harvested for H&E staining to examine histopathological changes.

### In vivo therapeutic evaluation

Tumor-bearing mice (100 to 200 mm^3^) were randomized into 8 groups (*n* = 5): saline, ALA-PDT, FCA-PDT, RT, ALA-RT, FCA-RT, ALA-PDT-RT, and FCA-PDT-RT. On days 0, 2, and 4, mice received tail vein injections of saline, ALA (1 mg/ml, 50 μl) or FCA (ALA-equivalent, 1 mg/ml, 50 μl). On days 1, 3 and 5, 660-nm laser irradiation (600 mW for 10 min) and/or 4-Gy x-rays were delivered according to group allocation. Tumor length (*a*), width (*b*), and body weight were recorded every other day for 12 d, and tumor volume was calculated as *V* = *a* × *b*^2^ / 2. At the end point, tumors and major organs were harvested for weighing, photography, and histological or immunohistochemical staining (H&E, TUNEL, CD3, CD8, CD31, and PpIX).

For intratumoral ROS analysis, tumors were digested into single-cell suspensions, stained with DCFH-DA, and analyzed by flow cytometry.

### Statistical analysis

All experiments were independently repeated at least 3 times. Data are presented as the means ± SD. Statistical analyses were performed using GraphPad Prism software (version 9.0). Comparisons between 2 groups were analyzed using a 2-tailed unpaired Student’s *t* test. Comparisons among multiple groups were analyzed by one-way analysis of variance (ANOVA), followed by Tukey’s test for post hoc multiple comparisons. Figures (including bar graphs and line charts) were generated using Origin software (version 2023). Data points in the graphs represent the mean, and error bars represent the SD. A *P* value of less than 0.05 was considered statistically significant, with *P < 0.05, **P < 0.01, and ***P < 0.001.

## Data Availability

The datasets used and analyzed during the current study are available from the corresponding author on reasonable request.
