# Supplementary material for: Dual Sensitization Enables Synergistic Photodynamic Therapy and Radiotherapy for Breast Cancer
Source: Research (Wash D C). 2026 Feb 6;9:1114. doi: 10.34133/research.1114 (PMC12876564; doi:10.34133/research.1114)
Supplement: Supplementary 1 — Figs. S1 to S22 [file research.1114.f1.pdf]

Supporting Information

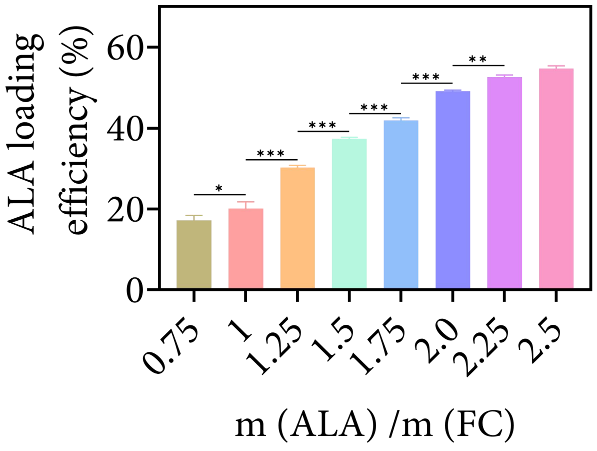

Figure S1 ALA loading efficiency in FCA.

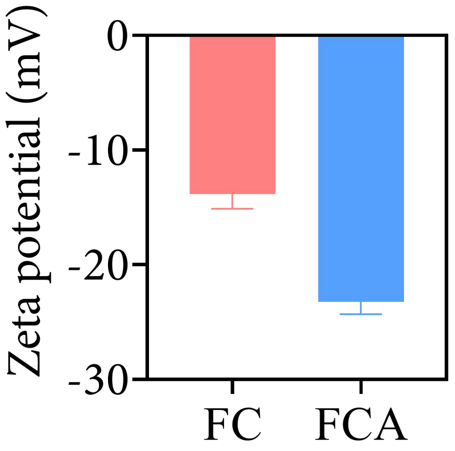

Figure S2 Zeta potentials of FC and FCA.

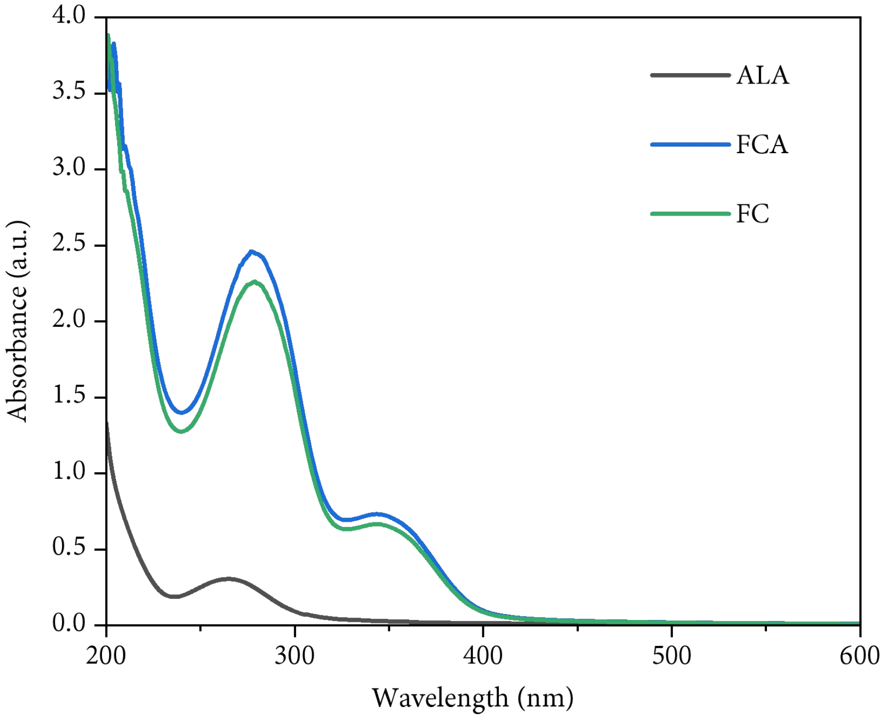

Fig. S3 UV-vis absorption spectrum of ALA、FC and FCA

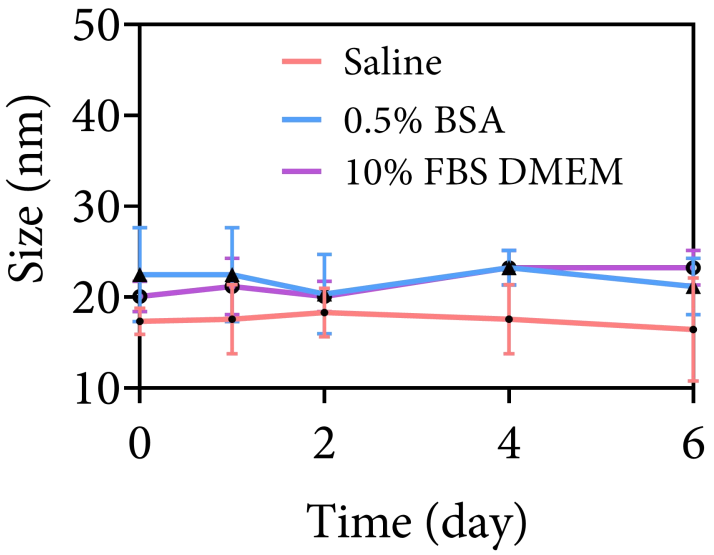

Fig. S4 Hydrodynamic diameter variation of FCA in saline, 0.5% BSA, and DMEM containing 10% FBS over 1-6 days.

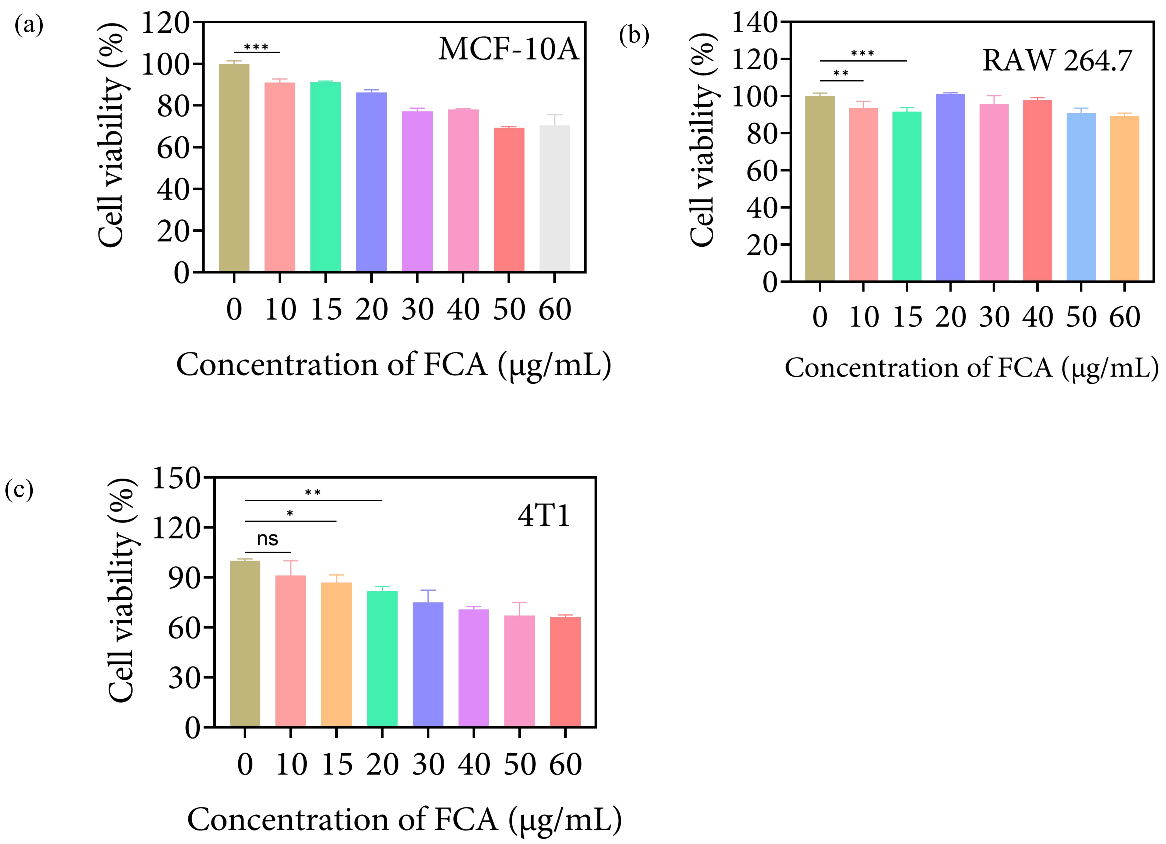

Figure S5 (a–c) Cell viability of MCF-10A, RAW264.7, and 4T1 cells after 24 h incubation with FCA at 0, 10, 15, 20, 30, 40, 50, and 60 μg/mL.

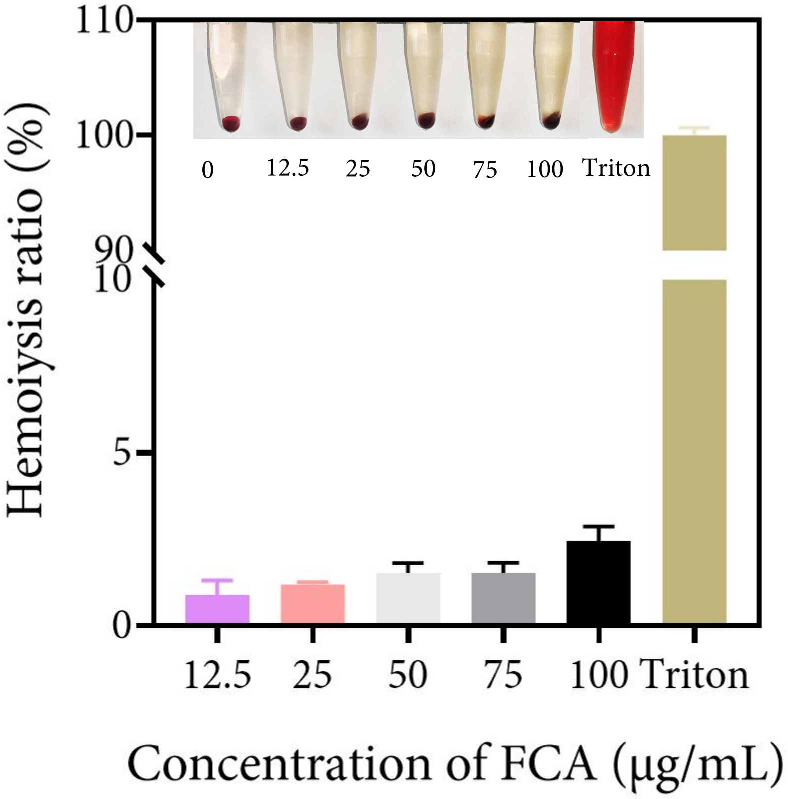

Figure S6 Hemolysis rate of FCA at different concentrations.

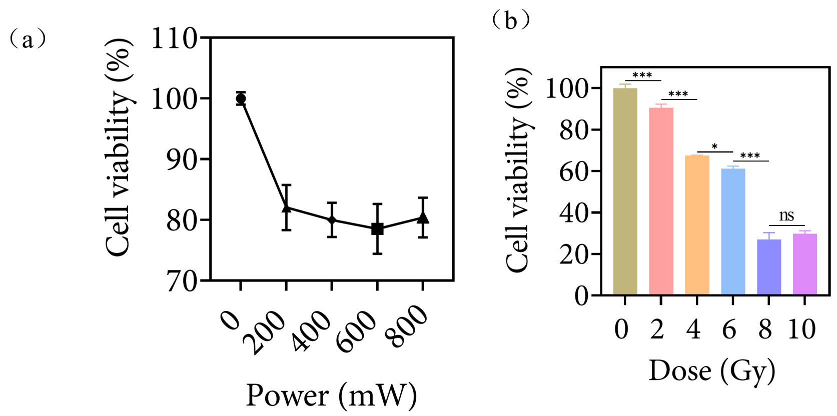

Figure S7 (a) Effect of laser power (0, 200, 400, 600, 800 mW) on 4T1 cell viability at an FCA concentration of 20 μg/mL; (b) Effect of radiation dose (0, 2, 4, 6, 8, 10 Gy) on 4T1 cell viability under 20 μg/mL FCA and 600 mW irradiation.

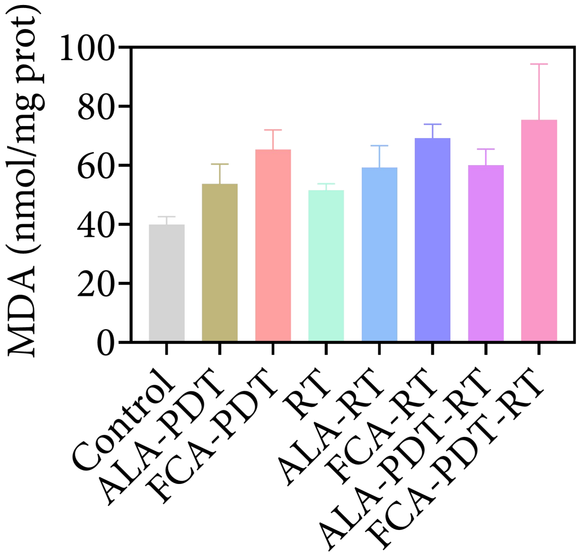

Figure S8 Intracellular malondialdehyde (MDA) levels across treatment groups.

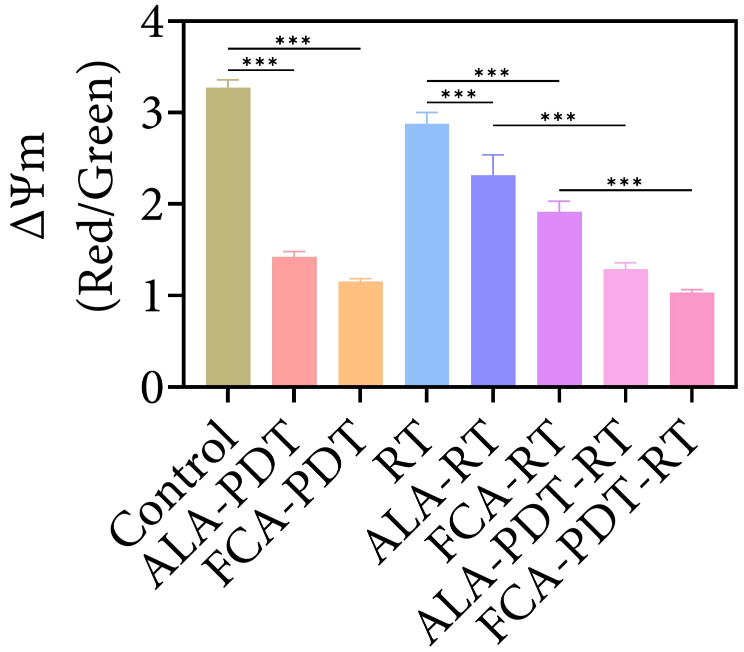

Figure S9 Effects of different treatments on mitochondrial membrane potential (ΔΨm) in 4T1 cells; JC-1 assay showing red/green fluorescence intensity ratios.

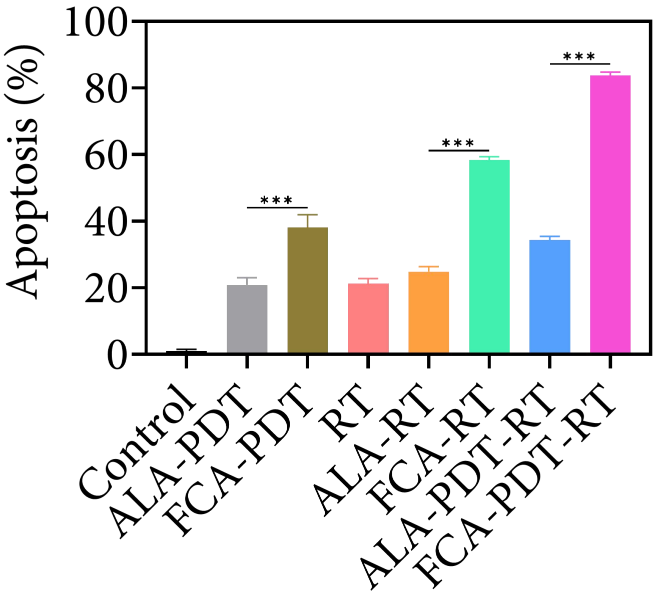

Figure S10 Quantification of apoptosis rates of 4T1 cells under different treatments.

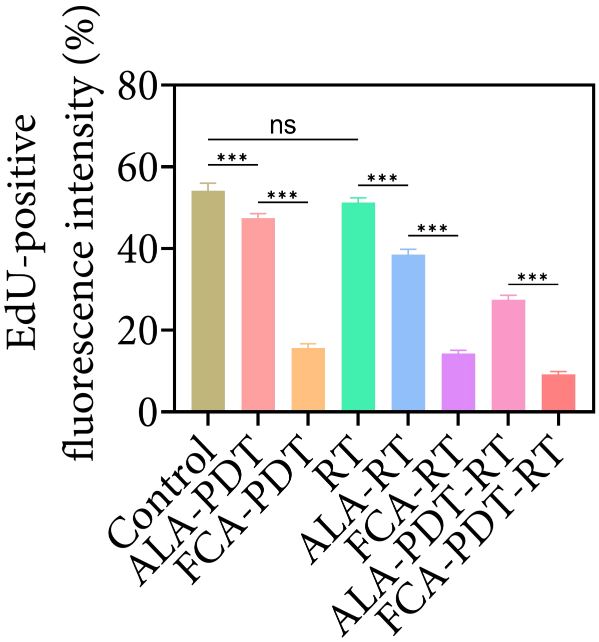

Figure S11 Quantitative analysis of EdU-positive fluorescence intensity in 4T1 cells for each treatment group.

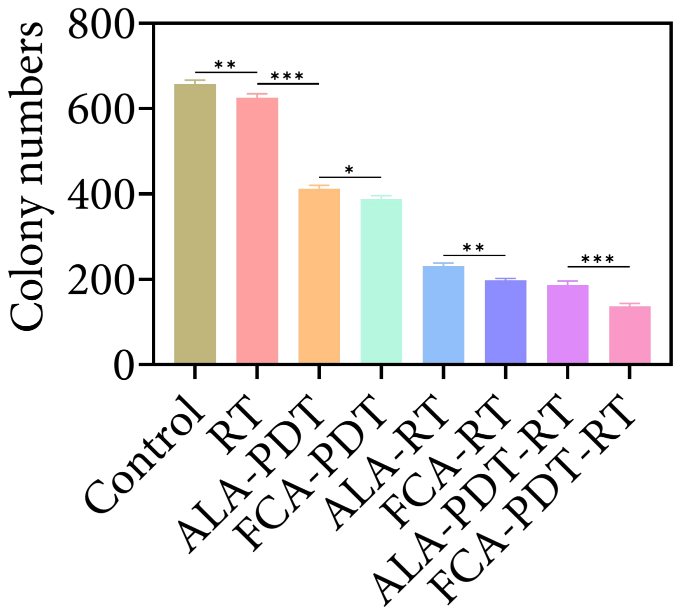

Figure S12 Quantitative analysis of colony formation for each treatment group.

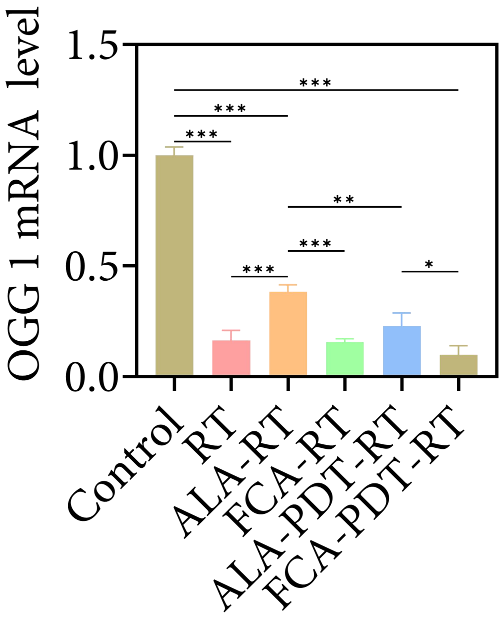

Figure S13 qRT-PCR analysis OGG1 mRNA expression.

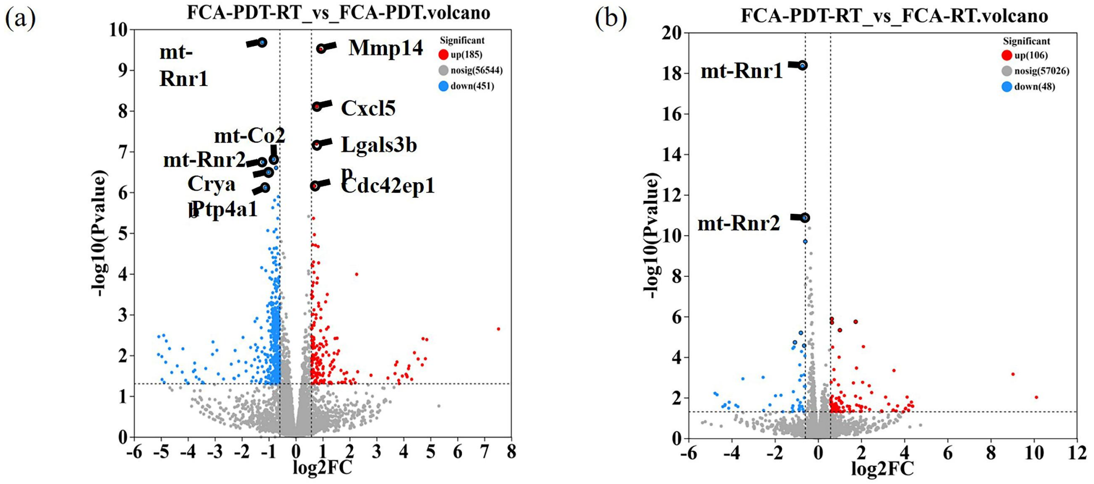

Figure S14 Differential gene expression analysis between FCA-mediated combination therapy and monotherapies.(a) Volcano plot (FCA-PDT-RT vs. FCA-PDT): red dots indicate significantly upregulated genes, blue dots indicate significantly downregulated genes.(b) Volcano plot (FCA-PDT-RT vs. FCA-RT): red dots indicate significantly upregulated genes, blue dots indicate significantly downregulated genes.

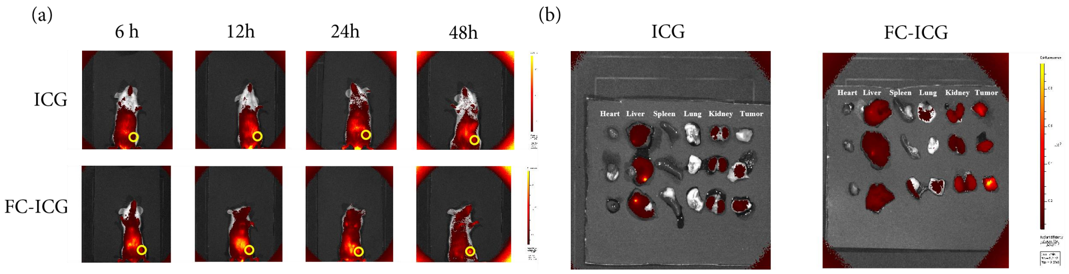

Figure S15 FC modification enhances tumor accumulation of ICG.(a) In vivo fluorescence images at 6, 12, 24, and 48 h after tail-vein injection of free ICG or FC-ICG (red fluorescence indicates ICG signal; tumor regions circled in yellow).(b) Ex vivo fluorescence images of heart, liver, spleen, lung, kidney, and tumor at 48 h.

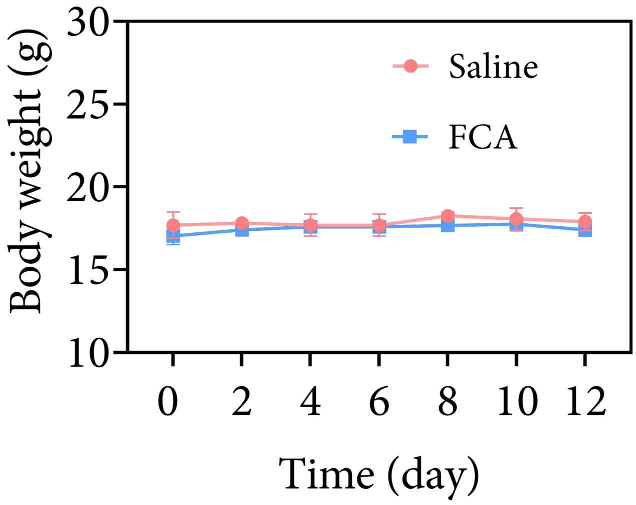

Fig. S16 Body weight changes of mice within 12 days after intravenous injection of normal saline or FCA via the tail vein (n = 3).

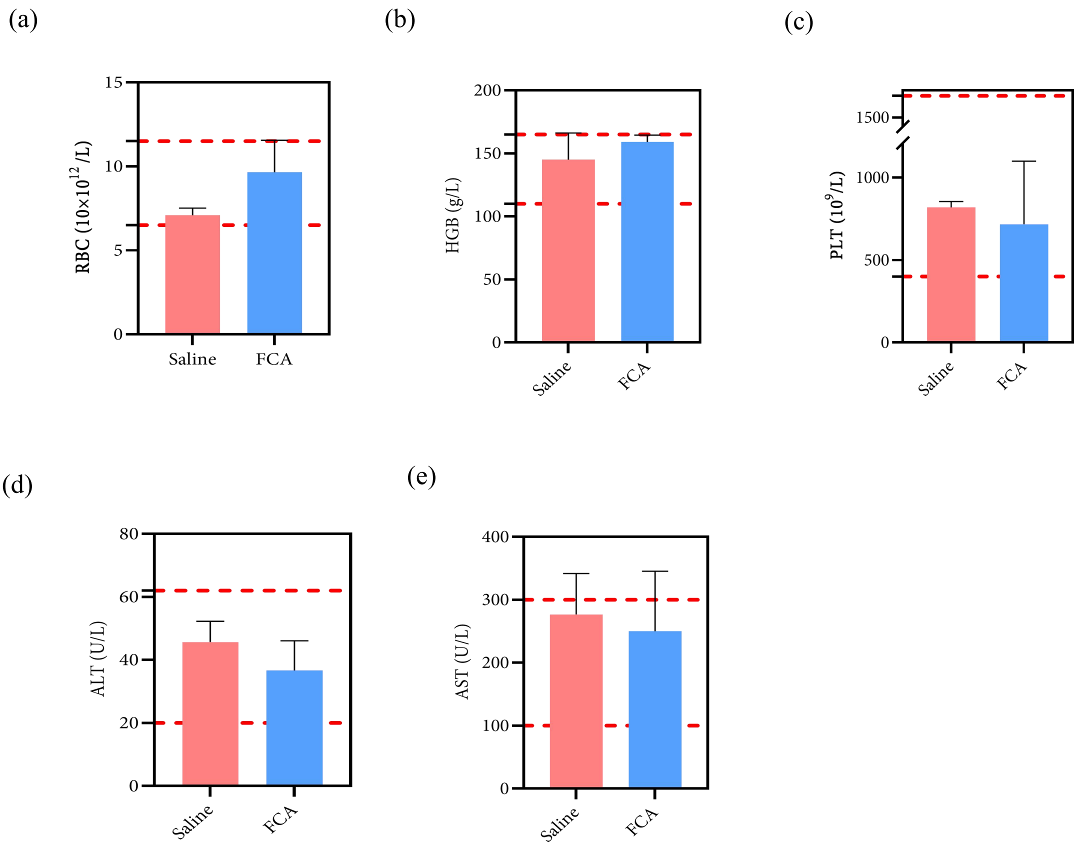

Fig. S17(a-e) Detection results of routine blood indicators in mice of the two groups on day 12 after administration

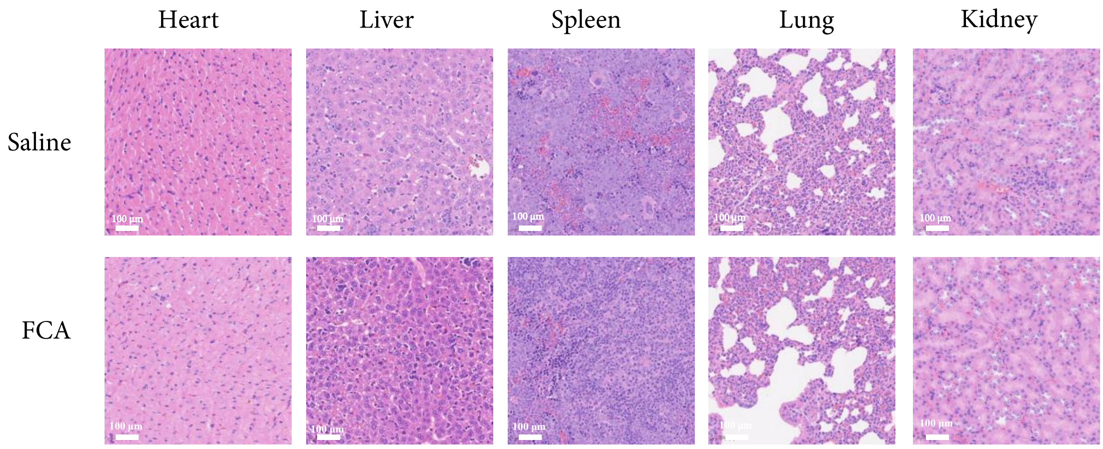

Fig. S18 H&E-stained sections of major organs (heart, liver, spleen, lung, kidney) on day 12 after administration (Scale bar: 100 μm).

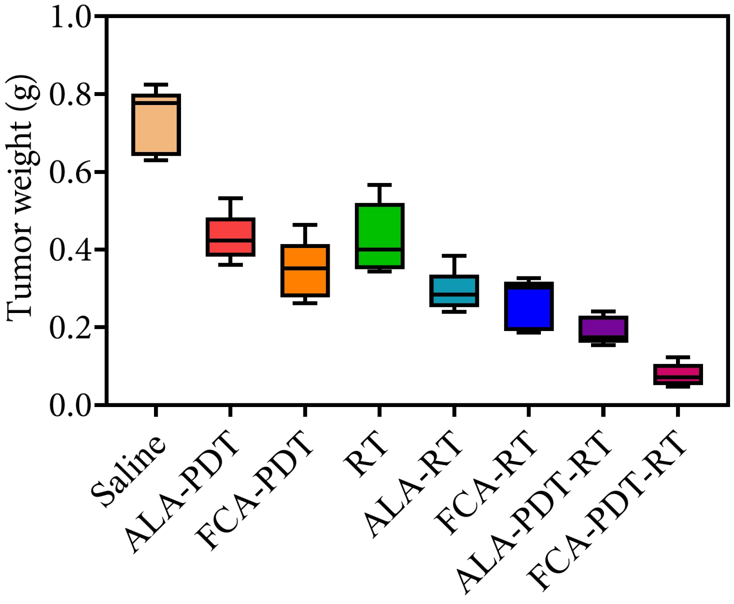

Figure S19 Tumor weights of tumor-bearing mice in different treatment groups.

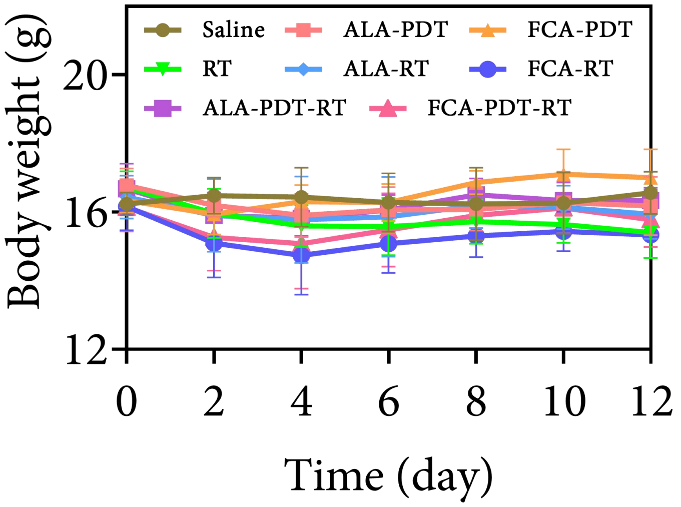

Figure S20 Body-weight trends of tumor-bearing mice during treatment.

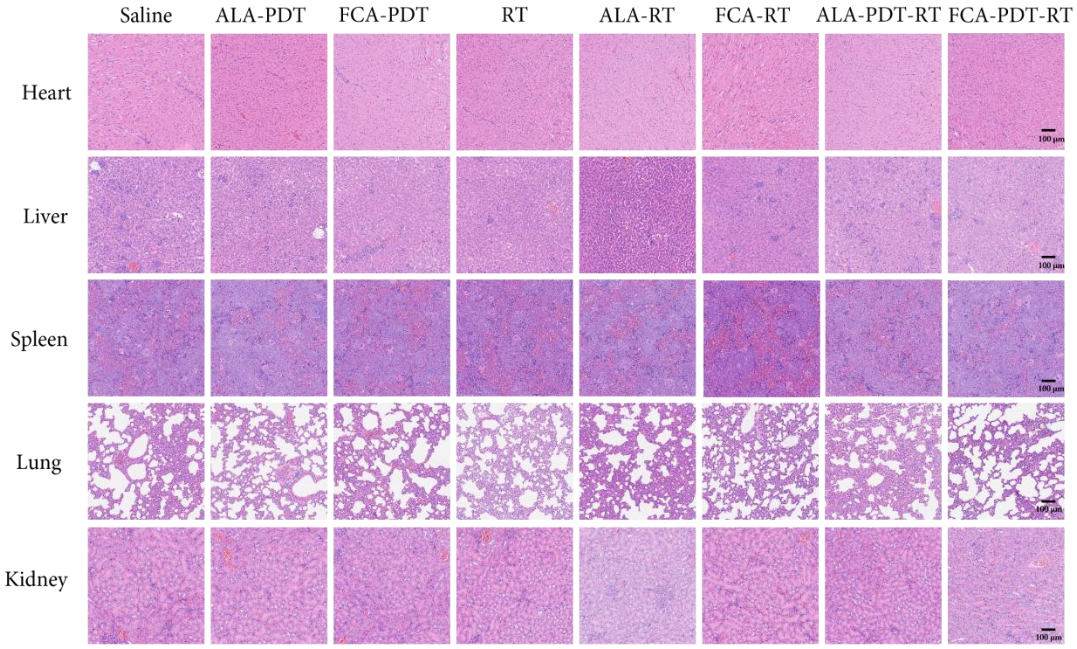

Figure S21 H&E of major organs (heart, liver, spleen, lung, kidney) from each treatment group; scale bar = 100 μm.

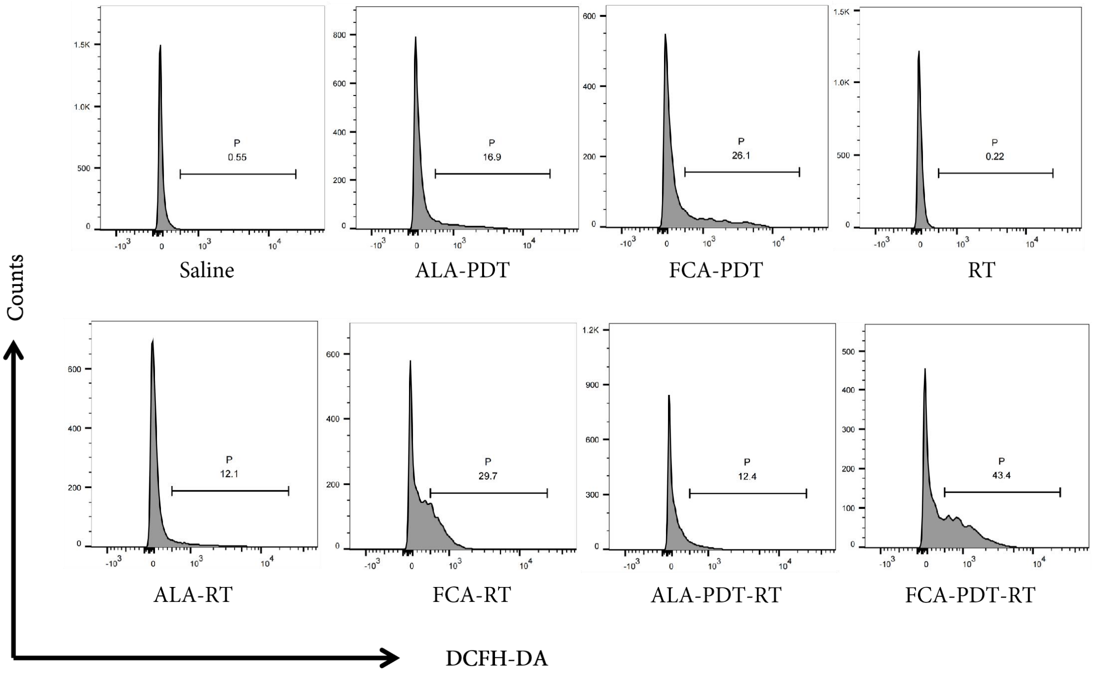

Figure S22 Flow-cytometric analysis of intratumoral ROS levels in different treatment groups.
